# Ryanodine Receptor 1 Is Dispensable for CD4^+^ T‐Cell Differentiation and Effector Function in Intestinal Inflammation Models

**DOI:** 10.1002/eji.70252

**Published:** 2026-08-17

**Authors:** Sogol Dostiar Tabrizi, Mikolaj Nawrocki, Tanja Bedke, Marius Böttcher, Friederike Stumme, Melina Birus, Franziska Möckl, Lola Hernandez, Björn‐Phillip Diercks, Nicola Gagliani, Dmitri Lodygin, Alexander Flügel, Andreas H. Guse, Hans‐Willi Mittrücker, Samuel Huber

**Affiliations:** ^1^ Section of Molecular Immunology and Gastroenterology, I. Department of Medicine University Medical Center Hamburg‐Eppendorf Hamburg Germany; ^2^ Hamburg Center for Translational Immunology University Medical Center Hamburg‐Eppendorf Hamburg Germany; ^3^ Department of Immunology University Medical Center Hamburg‐Eppendorf Hamburg Germany; ^4^ The Calcium Signaling Group, Department of Biochemistry and Molecular Cell Biology University Medical Center Hamburg‐Eppendorf Hamburg Germany; ^5^ Department of General, Visceral and Thoracic Surgery University Medical Center Hamburg‐Eppendorf Hamburg Germany; ^6^ Institute For Neuroimmunology and Multiple Sclerosis Research University Medical Center Göttingen Germany

**Keywords:** calcium signaling, CD4^+^ T cells, CD4^+^ T‐cell differentiation, intestinal inflammation, ryanodine receptor 1

## Abstract

T‐cell receptor (TCR) signaling is necessary for the activation and differentiation of CD4^+^ T cells. Calcium (Ca^2+^) signaling is essential for this process, and the complexity of Ca^2+^ channels presents a potential therapeutic target for modulating the strength of TCR signaling and further differentiation of CD4^+^ T cells. Nicotinic acid adenine dinucleotide phosphate (NAADP) is a potent Ca^2+^‐mobilizing second messenger that triggers Ca^2+^ release through ryanodine receptor 1 (RYR1) in T cells. While the molecular and biophysical properties of NAADP‐induced Ca^2+^ microdomains in T cells have been thoroughly investigated and the function of the NAADP–HN1L/JPT2–RYR1 axis has been supported in T‐cell activation and proliferation, its role in intestinal inflammation in vivo remains to be elucidated. In this study, we generated a conditional knockout mouse with *Ryr1* deleted in αβ T cells to investigate the functional relevance of RYR1 signaling in CD4^+^ T cells. *Ryr1* deletion in CD4^+^ T cells decreased TCR‐induced Ca^2+^ microdomain formation, reduced peak Ca^2+^ amplitude, and delayed the initial velocity of global Ca^2+^ signaling in vitro. However, *Ryr1* expression in CD4^+^ T cells was dispensable for their pathogenicity in murine models of intestinal inflammation. Thus, *Ryr1* expression in CD4^+^ T cells plays a redundant role in intestinal inflammation.

## Graphical abstract

*Ryr1* deletion reduces Ca^2+^ microdomains and delays global Ca^2+^ signaling in naïve CD4^+^ T cells but does not impair their activation, proliferation, or differentiation in vitro. Despite altered Ca^2+^ signaling, *Ryr1* expression in CD4^+^ T cells was dispensable for their pathogenicity in murine models of intestinal inflammation.

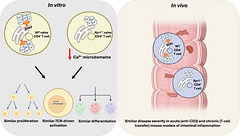

**Abbreviations**
EAE: experimental autoimmune encephalomyelitis
HN1L/JPT2: hematological and neurological expressed 1‐like protein/Jupiter microtubule‐associated homolog 2
IBD: inflammatory bowel disease
IP_3_
: d‐*myo*‐inositol 1,4,5‐trisphosphate
NAADP: nicotinic acid adenine dinucleotide phosphate
RYR1: ryanodine receptor 1
Tr1: Type 1 regulatory T‐cell
Treg: regulatory T‐cell

## 1 Introduction

Recognition of a cognate antigen via a T‐cell receptor (TCR) is a hallmark of the adaptive immune system, which initiates a complex signaling network that, depending on the composition of the inflammatory milieu, directs the immune response through T‐cell differentiation [1, 2]. Not only does the presence of specific cytokines in the inflammatory milieu influence the fate of CD4^+^ T cells, but the qualitative and quantitative characteristics of TCR signals also play a crucial role in directing CD4^+^ T‐cell differentiation [3, 4]. A critical component of the signaling network downstream of TCR activation is calcium (Ca^2+^) signaling [5, 6]. The intricate interplay of secondary messengers, Ca^2+^ channels, and ion pumps that regulate Ca^2+^ fluxes between cellular compartments generates spatiotemporal patterns that control essential cellular events following TCR activation. The multifaceted nature of Ca^2+^ signaling in T cells, therefore, presents a potentially attractive therapeutic target for fine‐tuning adaptive immune responses and controlling pathological chronic inflammatory conditions [6].

Recognition of antigen by the TCR leads to a rapid synthesis of Ca^2+^‐mobilizing secondary messengers d‐*myo*‐inositol 1,4,5‐trisphosphate (IP_3_), nicotinic acid adenine dinucleotide phosphate (NAADP), and cyclic ADP‐ribose (cADPR), which induce Ca^2+^ release from intracellular stores through specific Ca^2+^ channels [6]. Within milliseconds following TCR activation, Ca^2+^ microdomains at plasma membrane‐endoplasmic reticulum junctions are formed and culminate in the vigorous release of Ca^2+^ from the endoplasmic reticulum [7, 8]. The decrease in Ca^2+^ concentration within the lumen of the endoplasmic reticulum is sensed by stromal interaction molecules (STIM1 and STIM2), which open plasma membrane Ca^2+^ release‐activated Ca^2+^ channels, allowing extracellular Ca^2+^ influx into the cytosol during the process of store‐operated Ca^2+^ entry (SOCE) [9, 10]. Molecular mechanisms involved in the formation of microdomains and their functional relevance have recently been a subject of thorough investigation [8, 11]. The key second messenger mediating the development of Ca^2+^ microdomains upon TCR activation is NAADP [7, 12]. NAADP is synthesized within milliseconds following antigen recognition by the TCR, and then NAADP binds to the NAADP receptor hematological and neurological expressed 1‐like protein (HN1L)/Jupiter microtubule‐associated homolog 2 (JPT2) and releases Ca^2+^ from the endoplasmic reticulum via ryanodine receptor 1 (RYR1) [13]. Direct physical interaction between HN1L/JPT2 and RYR1 has not been demonstrated in vitro. In other cell types, HN1L/JPT2 also acts on acidic vesicles via two pore channels (TPC1 and TPC2) [14]. Importantly, it has been reported that Ca^2+^ microdomains not only represent a stage of the Ca^2+^ signaling cascade but also have specific physiological functions, such as in T‐cell migration, as well as in the trafficking of intracellular vesicles and membrane receptors [11, 15, 16].

The functional relevance of the NAADP–HN1L/JPT2–RYR1 axis has been proven to play an important role in T‐cell activation and differentiation in vitro and in vivo [13, 17, 18, 19, 20]. Small molecule antagonists of NAADP signaling impair activation and proliferation in vitro in a Ca^2+^‐dependent manner [19, 20]. Moreover, antagonizing NAADP skews T‐cell differentiation into a more regulatory phenotype in vitro and in vivo [19]. Deletion of the NAADP receptor HN1L/JPT2 resulted in decreased formation of Ca^2+^ microdomains as well as global Ca^2+^ signals in T cells [13]. In vivo studies showed that both antagonizing NAADP with the small molecule antagonist BZ194 and inhibiting ryanodine receptors with dantrolene ameliorate the activity of the experimental autoimmune encephalomyelitis (EAE) murine model of multiple sclerosis [17, 18, 21]. Studying the role of RYR1 was limited by the fact that the missense mutation of the channel is particularly important for the function of striated muscle and is therefore perinatally lethal [22]. A recent study used fetal liver chimeras to show that the deletion of *Ryr1* in hematopoietic cells decreases the severity of symptoms in EAE, and cellular analysis of these mice showed a decreased percentage of CD25^+^ and CD69^+^ encephalitogenic (activated) T cells in the spinal cord of these mice compared to wild‐type mice (Lécuyer et al. unpublished data). Likewise, the conditional deletion of *Ryr1* in T cells confirmed this finding (Lécuyer et al. unpublished data). In contrast, mice carrying a gain‐of‐function RYR1‐p.R163 mutation develops a faster and more severe EAE course than wild‐type mice [21].

Likewise, Trans‐Ned‐19, an NAADP antagonist, promotes the production of IL‐10 by effector cells, increases their suppressive capacity, and induces the transdifferentiation of Th17 cells into Tr1 cells, both in vitro and in vivo [19]. However, although the function of the NAADP–HN1L/JPT2–RYR1 axis has been proven in T‐cell activation and proliferation, its role in CD4^+^ T‐cell differentiation and intestinal inflammation remains to be elucidated.

Inflammatory bowel disease (IBD) is a chronic immune‐mediated disease with a multifactorial etiology including genetic predisposition, environmental factors, and a dysregulated T‐cell response [23, 24, 25]. Differentiation of CD4^+^ Th cells, particularly the balance between proinflammatory Th17 and Th1 cells and suppressive Foxp3^+^ T regulatory cells (Tregs) and IL‐10‐producing Tr1 cells, determines inflammatory activity and tissue damage [26, 27, 28, 29, 30]. Ca^2+^ signaling plays a critical role in the pathogenesis of IBD. Specifically, Ca^2+^ signaling in T cells was reported to control the severity of intestinal inflammation in murine models of IBD. T‐cell‐specific deletion of components of SOCE leads to decreased TCR‐induced Ca^2+^ signaling in T cells, reduced production of inflammatory cytokines, and alleviation of tissue damage in murine models of IBD [31, 32, 33, 34]. Moreover, inhibitors of calcineurin, a Ca^2+^‐dependent phosphatase required for activation of NFAT transcription factors, are recommended for steroid‐refractory cases of ulcerative colitis [35, 36]. Given the role of the NAADP–HN1L/JPT2–RYR1 axis in T‐cell function in neuroinflammation, as well as the importance of Ca^2+^ signaling in IBD, we aimed to investigate the role of RYR1 in T cells during intestinal inflammation.

## 2 Results

### 2.1 Generation and Validation of Αβ T‐Cell‐Specific RYR1 Conditional Knockout Mouse Line

Investigation of the role of RYR1 in T‐cell function using knockout mice was limited by the perinatal lethality of the missense mutation of *Ryr1* [22]. Previous studies delivered important mechanistic insights regarding the NAADP–HN1L/JPT2–RYR1 axis using chemical NAADP and RYR antagonists or in vitro generated CRISPR–Cas9 knockouts [13, 19, 20, 21, 37]. Nevertheless, these methods are prone to off‐target effects and do not allow for a specific evaluation of RYR1's role in T‐cell function in vivo.

**FIGURE 1:**
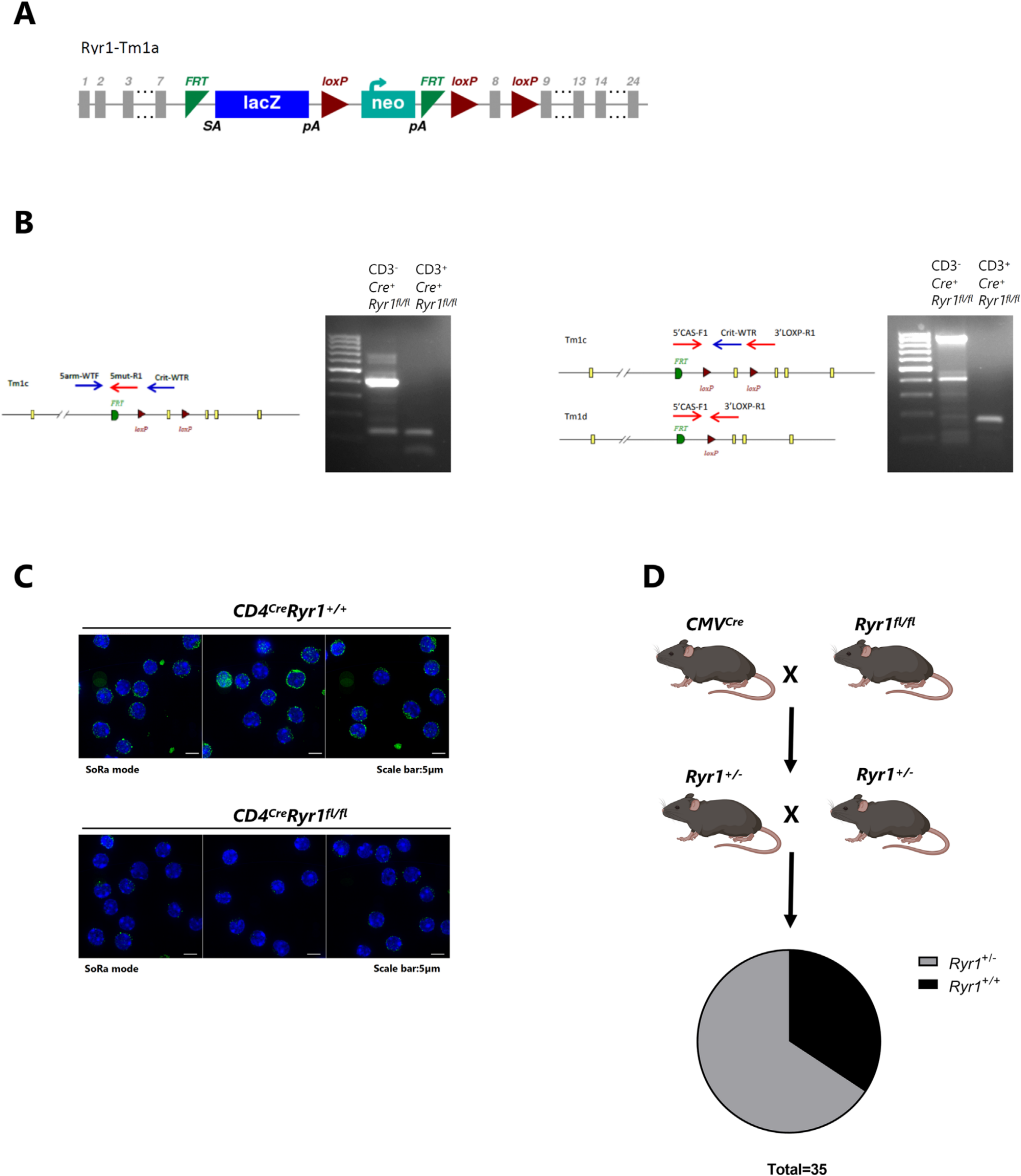
Generation and validation of the *CD4^cre^Ryr1* conditional knockout mouse line. (A) *Ryr1* floxed mice were generated by crossing conditional‐ready *Ryr1* ^tm1a(EUCOMM)^ mice with a Flp recombinase‐expressing strain, resulting in a *tm1c* allele with exon 8 flanked by loxP sites (scheme reproduced from the EUCOMM program). (B) Schematic of the *Ryr1^tm1c^* locus and its Cre‐mediated recombination into the *Ryr1* ^tm1d^ allele (scheme reproduced from the EUCOMM programs). CD3^+^ and CD3^−^ cells were sorted by FACS from a *CD4^cre^Ryr1^fl^* ^/^ *^fl^* mouse, and genomic DNA was analyzed by PCR to confirm recombination. Left: The tm1c allele produces a 461 bp band in nonrecombined CD3^−^ cells and a 125 bp mutant band in both CD3^+^ and CD3^−^ cells. Right: The tm1d allele yields a 174 bp product, whereas the tm1c allele generates a larger product that includes the critical region. (C) Immunofluorescence staining of CD4^+^ T cells from *CD4^cre^Ryr1* ^+/+^ and *CD4^cre^Ryr1^fl^* ^/^ *^fl^* mice using an anti‐RYR (F‐1) antibody. Each field of view represents one mouse. Representative of two independent experiments. (D) Total *Ryr1* knockout mice were generated by crossing *Ryr1^tm1c^* conditional knockout mice with a *CMV^Cre^ line*. Breeding outcomes indicate that complete loss *of Ryr1* causes embryonic or perinatal lethality.

To overcome these limitations, we generated an *Ryr1* conditional knockout mouse line. We used a conditional ready *Ryr1^tm1a^* line, in which the *Ryr1* allele contains a gene trap cassette (lacZ and neomycin), loxP sites, and FRT sites flanking exon 8. In the first step, *Ryr1^tm1a^* mice were crossed with FLP recombinase mice to remove the gene trap cassette and generate a functional conditional allele (*Ryr1^tm1c^*). In the next step, the resulting *Ryr1* ^1c^ mice were crossed with *CD4^cre^* mice to generate T‐cell‐specific *Ryr1* knockout mice *Ryr1^tm1d^*). These mice were then further crossed to a triple reporter mouse expressing fluorescent proteins reporting for IFN‐γ [38], IL‐17A [29], and Foxp3 [39], resulting in *CD4^cre^Ryr1* ^+/+^ *IFN‐γ^katushka^IL‐17^eGFP^Foxp3^RFP^* and *CD4^cre^Ryr1^fl^* ^/^ *^fl^ IFN‐γ^katushka^IL‐17^eGFP^Foxp3^RFP^* mice (Figure 1A,B).

To validate the correct integration of the conditional allele and *Ryr1* knockout efficiency, we used different sets of primers for ^c^ *Ryr1^tm1c^* and *Ryr1^tm1d^*. PCR analysis of DNA from CD3^+^ and CD3^−^ cells sorted by FACS from *CD4^cre^Ryr1^fl^* ^/^ *^fl^* mice confirmed successful Cre‐mediated recombination (Figure 1B). Immunofluorescence staining with a pan‐RYR antibody confirmed the loss of ryanodine receptors in CD4^+^ T cells in *CD4^cre^Ryr1^fl^* ^/^ *^fl^* mice at the protein level (Figure 1C). Moreover, to further validate the accuracy of the floxed allele, we crossed *Ryr1^fl^* ^/^ *^fl^* mice with *CMV^cre^* mice and observed no viable *Ryr1* ^−^ *^/^* ^−^ offspring, confirming embryonic lethality due to global *Ryr1* deletion (Figure 1D).

### 2.2 Deletion of RYR1 in T Cells Impairs Formation of Ca^2+^ Microdomains and Global Ca^2+^ Signaling Upon TCR Stimulation

The endoplasmic reticulum‐NAADP signaling model in T cells explains NAADP‐evoked Ca^2+^ signaling. In this model, following TCR stimulation and NAADP formation, NAADP binds to its binding protein HN1L/JPT2, and the complex interacts with RYR1, leading to the formation of Ca^2+^ microdomains [40].

**FIGURE 2:**
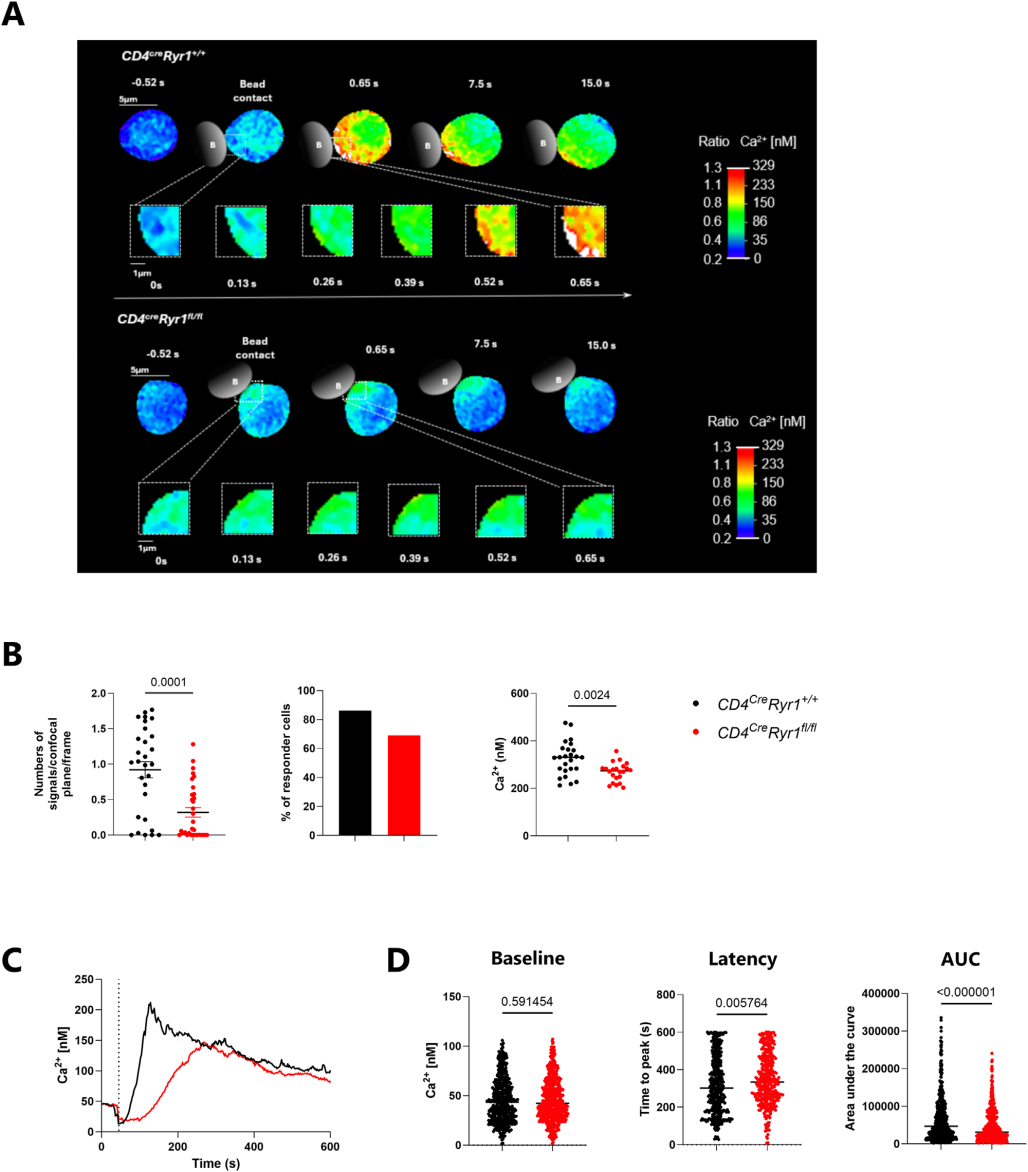
*Ryr1* deficiency impairs early Ca^2+^ signaling in naïve CD4^+^ T cells upon TCR/CD28 stimulation. (A) Representative images show early Ca^2+^ microdomain formation in a *CD4^cre^Ryr1* ^+/+^ T‐cell (top) and a *CD4^cre^Ryr1^fl^* ^/^ *^fl^* T‐cell (bottom) following stimulation with beads coated with anti‐CD3 and anti‐CD28 mAbs. A schematic illustrates the bead–cell contact site. Magnified views highlight regions near the bead interaction site. (B) Quantification of Ca^2+^ microdomains formed in *CD4^cre^Ryr1* ^+/+^ (*n* = 29) and *CD4^cre^Ryr1^fl^* ^/^ *^fl^* (*n* = 32) naïve CD4^+^ T cells in the first 15 s after contact with beads coated with anti‐CD3 and anti‐CD28 mAbs. Parameters analyzed included the number of signals detected per confocal plane per frame, the proportion of cells responding, and the Ca^2+^ signal amplitude. Data are presented as the mean ± SEM and were collected from seven independent experiments. Statistical analysis was performed using the unpaired Mann‒Whitney *U* test. (C) Global Ca^2+^ signaling in *CD4^cre^Ryr1* ^+/+^ (*n* = 496) and *CD4^cre^Ryr1^fl^* ^/^ *^fl^* (*n* = 536) naïve CD4^+^ T cells. Cells were loaded with Fura‐2 and stimulated with anti‐CD3 mAb, followed by ratiometric Ca^2+^ imaging for 10 min. Data represent the mean kinetics of cytosolic Ca^2+^ concentration over time. (D) Quantification of global Ca^2+^ responses, including baseline Ca^2+^ concentration, latency to peak, and area under the curve (AUC). Data are shown as the mean ± SEM collected from three independent experiments. Statistical significance was determined by the unpaired Mann‒Whitney *U* test.

Upon TCR/CD28 stimulation, naïve CD4^+^ T cells lacking RYR1 showed a reduced number of Ca^2+^ microdomains per cell, lower Ca^2+^ signal amplitude, and decreased proportion of responder cells within the first 15 s of T‐cell activation compared to wild‐type controls (Figure 2A,B). To assess whether reduced Ca^2+^ microdomain formation was reflected in global cytosolic Ca^2+^ dynamics, we measured the free cytosolic Ca^2+^ concentration in naïve CD4^+^ T cells for 10 min after stimulation with soluble anti‐CD3 mAb (Figure 2C,D). The baseline Ca^2+^ concentration was similar in wild‐type and *Ryr1‐*deficient cells. However, the latency to reach the peak calcium concentration upon TCR stimulation was significantly longer in *Ryr1*‐deficient T cells than in wild‐type T cells. This delay was further reflected in the overall lower area under the curve in the knockout cells over a 10‐min observation period (Figure 2C,D). Thus, deletion of *Ryr1* in CD4^+^ T cells reduced TCR‐induced microdomain formation, resulting in a slower rise in the intracellular calcium concentration and, consequently, reduced calcium entry. These findings are consistent with previous reports using *RYR1*‐knockdown Jurkat T cells [7], as well as results obtained with T cells derived from *Ryr1* ^−/−^ fetal liver chimeric mice, which exhibited impaired microdomain formation [7, 12].

### 2.3 T‐Cell Specific Deletion of *Ryr1* Does Not Impact CD4^+^ T‐Cell Composition in Steady State

**FIGURE 3:**
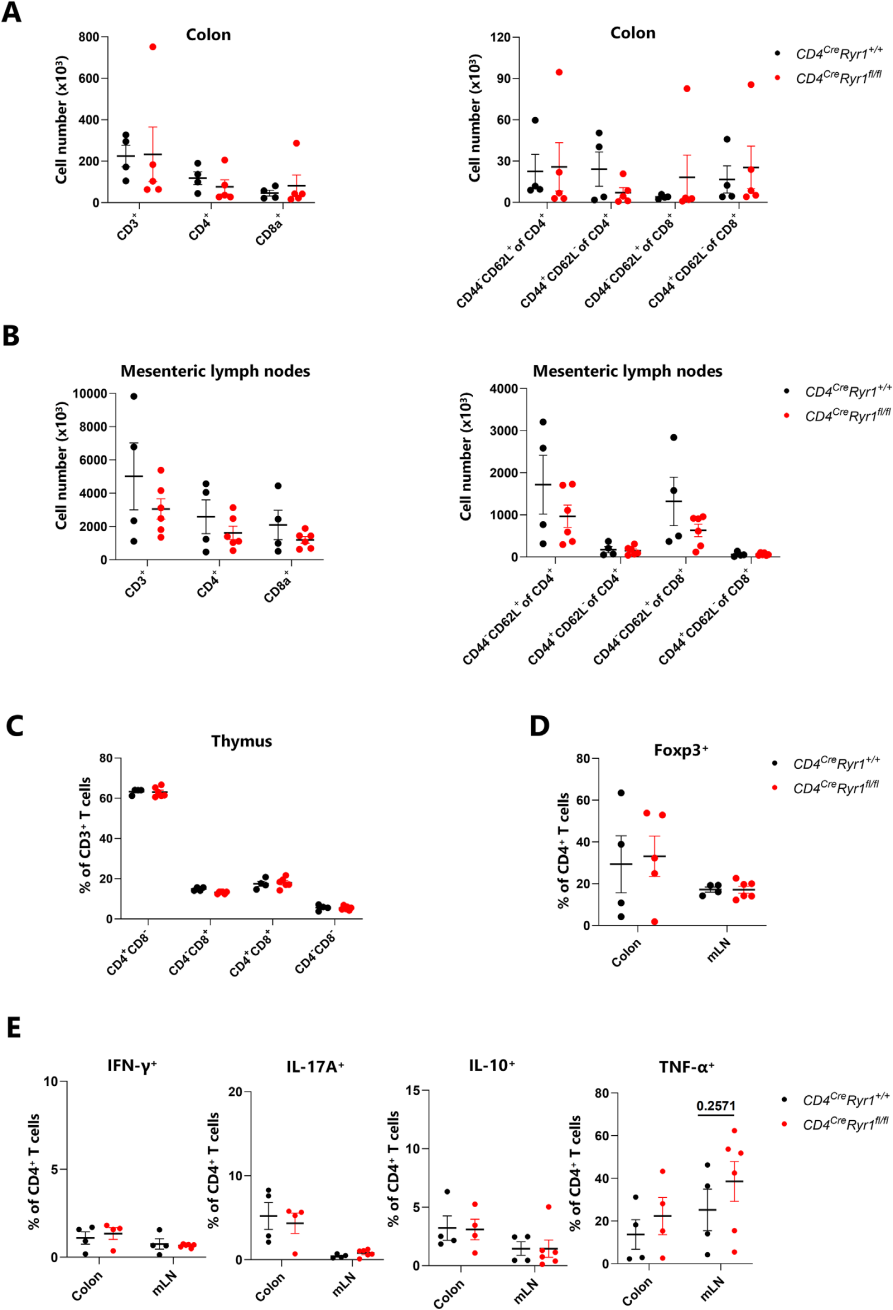
T‐cell compartment at steady state upon CD4Cre‐driven deletion of *Ryr1*. (A, B) Absolute numbers of CD3^+^, CD4^+^, and CD8^+^ T cells, as well as naïve (Foxp3^−^CD44^−^CD62L^+^) and effector (Foxp3^−^CD44^+^CD62L^−^) CD4^+^ and CD8^+^ T cells, were measured in the (A) colon and (B) mesenteric lymph nodes of *CD4^cre^Ryr1* ^+/+^ and *CD4^cre^Ryr1^fl^* ^/^ *^fl^* mice. (C) Frequencies of CD3^+^ CD4^+^, CD8^+^, double‐positive (DP), and double‐negative (DN) thymocytes in *CD4^cre^Ryr1* ^+/+^ and *CD4^cre^Ryr1^fl^* ^/^ *^fl^* mice. (D) Frequencies of Tregs in the colon and mesenteric lymph nodes (mLN) of *CD4^cre^Ryr1* ^+/+^ and *CD4^cre^Ryr1^fl^* ^/^ *^fl^* mice. (E) Cytokine production (IFN‐γ, IL‐17A, TNF‐α, and IL‐10) by CD4^+^ T cells isolated from the colon and mesenteric lymph nodes of *CD4^cre^Ryr1* ^+/+^ and *CD4^cre^Ryr1^fl^* ^/^ *^fl^* mice following stimulation with PMA and ionomycin. Each group included four to six mice pooled from two independent experiments. Mice were 7–23 weeks old at the time of analysis. All data are presented as the mean ± SEM. Statistical comparisons were performed using the unpaired Mann‒Whitney *U* test.

To determine whether RYR1 plays a role in T‐cell development and normal immune composition, we analyzed T‐cell populations and cytokine production in *CD4^cre^Ryr1* ^+/+^ and *CD4^cre^Ryr1^fl^* ^/^ *^fl^* mice under steady‐state conditions. Flow cytometry analysis of CD3^+^, CD4^+^, and CD8^+^ T cells from colon and mesenteric lymph nodes showed no significant differences in CD62L^+^CD44^low^ naïve and CD62L^−^CD44^high^ effector T‐cell numbers between *CD4^cre^Ryr1* ^+/+^ and *CD4^cre^Ryr1^fl^* ^/^ *^fl^* mice (Figure 3A,B). In the thymus, the frequencies of CD4^+^, CD8^+^, CD4^+^CD8^+^ (double positive), and CD4^−^CD8^−^ (double negative) thymocytes were comparable between groups (Figure 3C). Furthermore, Foxp3^+^ Treg cells were also present at similar frequencies between both groups in mesenteric lymph nodes and colon (Figure 3D). Upon ex vivo stimulation with phorbol 12‐myristate 13‐acetate (PMA) and ionomycin in the presence of monensin, the production of IFN‐γ, IL‐17A, IL‐10, and TNF‐α by CD4^+^ T cells was largely comparable between wild‐type and *CD4^cre^Ryr1^fl^* ^/^ *^fl^* mice. TNF‐α production in CD4^+^ T cells isolated from mesenteric lymph nodes was higher in conditional knockout mice than in wild‐type controls; nevertheless, this result did not reach statistical significance (Figure 3E). Thus, RYR1 is dispensable for the emergence of CD4^+^ T‐cell subpopulations in the steady state.

### 2.4 *Ryr1* Expression Shows a Trend Toward Higher Levels in Effector Subsets of CD4^+^ T Cells

**FIGURE 4:**
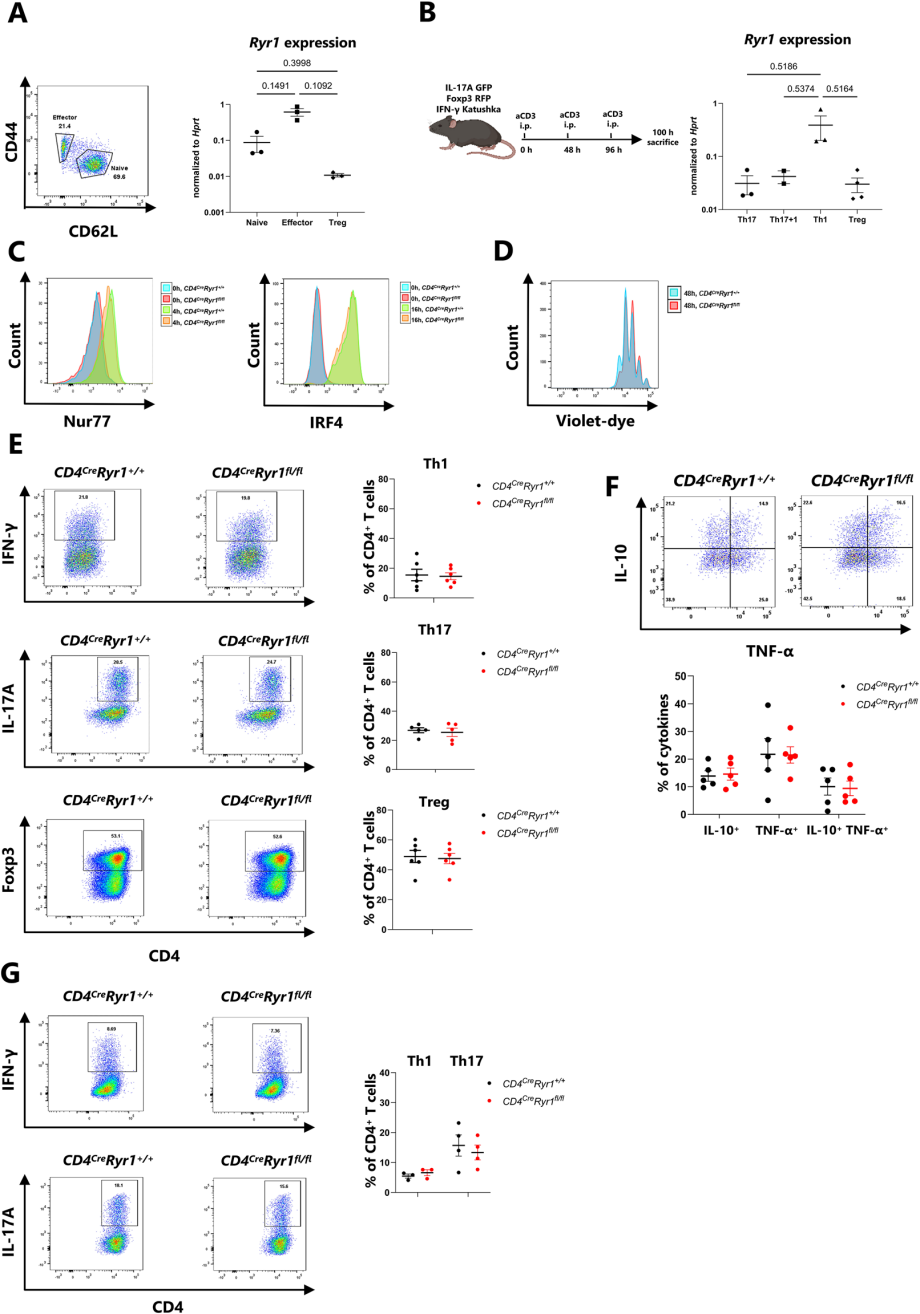
*Ryr1* expression and functional characterization in CD4^+^ T‐cell activation, proliferation, and in vitro differentiation. (A, B) Quantitative RT‐PCR analysis of *Ryr1* expression in (A) CD62L^+^CD44^low^ naïve and CD62L^−^CD44^high^ effector CD4^+^ T cells and Foxp3^+^ T regulatory cells (Tregs) from spleens of WT mice at steady state (three biological replicates per group) and (B) in Th1, Th17, Th1+17, and Tregs sorted using FACS from small intestines of *Foxp3^RFP^ IL‐17A^eGFP^ IFN‐γ^Katushka^* triple reporter mice after induction of intestinal inflammation by anti‐CD3 mAb administration (*n* = 2–4 biological replicates per group). Statistical analysis was performed on log‐transformed data using Welch's one‐way ANOVA followed by Dunnett's T3 multiple‐comparisons test. (C) Naïve CD4^+^ T cells from *CD4^cre^Ryr1* ^+/+^ and *CD4^cre^Ryr1^fl^* ^/^ *^fl^* mice were stimulated in vitro with anti‐CD3 and anti‐CD28 mAbs. The expression of the early TCR activation markers Nur77 and IRF4 was measured by FACS at 4 and 16 h post‐stimulation, respectively (*n* = 3 biological replicates per group). (D) Naïve CD4^+^ T cells from *CD4^cre^Ryr1* ^+/+^ and *CD4^cre^Ryr1^fl^* ^/^ *^fl^* mice were labeled with CellTrace Violet before stimulation with anti‐CD3 and anti‐CD28 mAbs. Proliferation was assessed by CellTrace Violet dye dilution via flow cytometry after 48 h (*n* = 3 biological replicates per group). (E) In vitro differentiation of naïve CD4^+^ T cells into Th1, Th17, and Treg cells. FACS‐sorted CD62L^+^CD44^low^ CD4^+^ T cells from *CD4^cre^Ryr1* ^+/+^ and *CD4^cre^Ryr1^fl^* ^/^ *^fl^* mice were cultured for 96 h under polarizing conditions. Cytokine expression was assessed after 4 h of restimulation with ionomycin and PMA. Representative dot plots and frequencies of IFN‐γ^+^ Th1 cells, IL‐17A^+^ Th17 cells, and Foxp3^+^ Treg cells are shown (*n* = 5–6 biological replicates per group). (F) In vitro restimulation of effector CD4^+^ T cells. FACS‐sorted CD62L^−^CD44^high^ effector CD4^+^ T cells from *CD4^cre^Ryr1* ^+/+^ and *CD4^cre^Ryr1^fl^* ^/^ *^fl^* mice were cultured with anti‐CD3 and anti‐CD28 mAbs in the presence of IL‐2 for 96 h. Cytokine expression was assessed after 4 h of restimulation with ionomycin and PMA. Representative dot plots and frequencies of effector CD4^+^ T cells producing IL‐10 and TNF‐α (*n* = 5 biological replicates per group) are shown. (G) In vitro differentiation of naïve CD4^+^ T cells into Th1and Th17 cells. FACS‐sorted CD62L^+^CD44^low^ CD4^+^ T cells from *CD4^cre^Ryr1* ^+/+^ *IL‐17A^eGFP^IFN‐γ^Katushka^* and *CD4^cre^Ryr1^fl^* ^/^ *^fl^ IL‐17A^eGFP^IFN‐γ^Katushka^* mice were cultured for 96 h under polarizing conditions. Cytokine expression was assessed without restimulation. Representative dot plots and frequencies of IFN‐γ^+^ Th1 cellsand IL‐17A^+^ Th17 cells are shown (*n* = 3–4 biological replicates per group). All experiments were independently performed at least three times. All data are presented as the mean ± SEM. Statistical comparisons were made using the unpaired Mann‒Whitney *U* test.

Next, we sought to determine the expression pattern of *Ryr1* in CD4^+^ T‐cell subsets. To this end, we sorted Foxp3^+^ Treg cells, CD62L^+^CD44^low^ naïve, and CD62L^−^CD44^high^ effector CD4^+^ T cells from the spleens of wild‐type mice under steady‐state conditions by FACS. Quantitative RT‐PCR analysis revealed that *Ryr1* expression tended to be higher in effector T cells than in naïve and regulatory T cells, although these differences did not reach statistical significance (Figure 4A). To further investigate *Ryr1* expression in effector subsets and Foxp3^+^ Treg cells, during intestinal inflammation, we induced transient intestinal inflammation by injecting anti‐CD3 mAb into *IFN‐γ^katushka^ IL‐17^eGFP^ Foxp3^RFP^* reporter mice, which express fluorescent proteins upon Foxp3, IL‐17A, or IFN‐γ expression. Using flow cytometry, we sorted IFN‐γ^Katushka+^ Th1, IL‐17A^eGFP+^ Th17, and IFN‐γ^Katushka+^ IL‐17A^eGFP+^ Th1/Th17 cells, as well as Foxp3^+^ Treg cells, and quantified *Ryr1* expression in these subsets. Among these subsets, *Ryr1* expression showed the highest expression in Th1 cells (Figure 4B).

Taken together, *Ryr1* expression showed a trend toward higher expression in effector CD4^+^ Th1 cells compared to other CD4^+^ T‐cell subsets.

### 2.5 *Ryr1* Deficiency Does Not Impair CD4^+^ T‐Cell Activation and Differentiation In Vitro

Ca^2+^ signaling is essential for regulating primary T‐cell activation, function, cytokine production, and proliferation [6, 41, 42, 43, 44], and disruption of Ca^2+^ signaling impairs the T‐cell response and function [32]. Therefore, we sought to investigate whether RYR1‐mediated Ca^2+^ flux plays a role in the activation and differentiation of CD4^+^ T cells.

Therefore, we next analyzed early activation markers in naïve CD4^+^ T cells following TCR stimulation. To this end, we isolated naïve CD4^+^ T cells from *CD4^cre^Ryr1* ^+/+^ and *CD4^cre^Ryr1^fl^* ^/^ *^fl^* mice and stimulated them in vitro with anti‐CD3 and anti‐CD28 mAbs. The transcription factors Nur77 and IRF4 are markers of T‐cell activation, and their expression is correlated with TCR signal strength [45, 46]. No significant difference in the upregulation of these markers was observed between *CD4^cre^Ryr1* ^+/+^ and *CD4^cre^Ryr1^fl^* ^/^ *^fl^* T cells (Figure 4C). In line with these findings, *Ryr1* deficiency did not change T‐cell proliferation under in vitro conditions (Figure 4D). Similar results were obtained under lower anti‐CD3 stimulation conditions (Figure S1). These results suggest that RYR1 is dispensable for early TCR signaling and initial T‐cell activation.

To determine whether RYR1 affects CD4^+^ T‐cell differentiation and cytokine production, we sorted CD62L^+^CD44^low^ naïve and CD62L^−^CD44^high^ effector CD4^+^ T cells by FACS from *CD4^cre^Ryr1* ^+/+^ and *CD4^cre^Ryr1^fl^* ^/^ *^fl^* mice and stimulated them in vitro. Naïve cells were polyclonally activated using anti‐CD3 and anti‐CD28 mAbs and cultured under Th1 (IL‐12, IL‐2, and anti–IL‐4 mAb), Th17 (IL‐6, TGF‐β, anti‐IL‐4 mAb, and anti‐IFN‐γ mAb), and Treg (TGF‐β, IL‐2) polarizing conditions to promote subset‐specific differentiation. Effector T cells were restimulated with anti‐CD3 and anti‐CD28 mAbs in the presence of IL‐2, without additional polarizing cytokines, to assess their cytokine production capacity. Cytokine production and transcription factor expression were assessed after 4 days using flow cytometry. For intracellular cytokine staining, cells were restimulated with PMA, ionomycin, and monensin. Flow cytometric analysis revealed no differences in the in vitro differentiation of naïve T cells or cytokine production by effector T cells (Figure 4E,F).

Since PMA and ionomycin bypass physiological Ca^2+^ signaling and may override the effects of impaired RYR1‐mediated Ca^2+^ release, we repeated the assays without restimulation. We crossed the *CD4^cre^Ryr1^fl^* ^/^ *^fl^* line with reporter mice expressing fluorescent proteins for IL‐17A and IFN‐γ. In line with previous findings, *Ryr1*‐deficiency did not alter IL‐17A or IFN‐γ expression under Th17‐ and Th1‐polarizing conditions, respectively (Figure 4G). These results indicate that RYR1 is not essential for CD4^+^ T‐cell differentiation or cytokine production in vitro.

In summary, these results show that *Ryr1* expression is upregulated in effector T cells, particularly in Th1 cells. However, *Ryr1* deficiency did not change early TCR activation, T‐cell proliferation, differentiation, or cytokine production in vitro.

### 2.6 *Ryr1* Deficiency Does Not Significantly Alter T‐Cell‐Mediated Acute and Chronic Intestinal Inflammation

Our in vitro data indicated that RYR1 is dispensable for the signaling cascade downstream of TCR activation as well as for T‐cell proliferation and differentiation into Th1, Th17, and Foxp3^+^ Treg cells. Nevertheless, *Ryr1* expression is upregulated in effector T cells, while naïve T cells express lower amounts. Furthermore, in vitro assays cannot fully reproduce the complexity of inflammatory responses in vivo. Therefore, we next aimed to investigate whether RYR1 signaling in T cells plays a role in vivo in the context of intestinal inflammation. Indeed, mucosal immunity is regulated by a balance between effector Th1 and Th17 cells, regulatory Foxp3^+^ T cells, and Foxp3^−^ T regulatory 1 (Tr1) cells and is shaped by complex interactions between immune cells, microbiota‐derived signals, cytokine networks, and environmental factors [47].

To evaluate the role of RYR1 signaling in T cells during acute intestinal inflammation, *CD4^cre^Ryr1* ^+/+^ and *CD4^cre^Ryr1^fl^* ^/^ *^fl^* mice were systemically administered anti‐CD3 mAb. This model is characterized by systemic T‐cell activation followed by global inflammatory responses with a cytokine storm. These responses promote Th17 cell differentiation and migration to the small intestine as well as the expansion of Foxp3^+^ Treg cells and Tr1 cells, which suppress Th17 cells via IL‐10 signaling [29, 30]. Repeated anti‐CD3 mAb injections result in intestinal inflammation, which primarily affects the upper small intestine and causes significant weight loss, recapitulating acute immune‐mediated tissue damage [30, 48].

**FIGURE 5:**
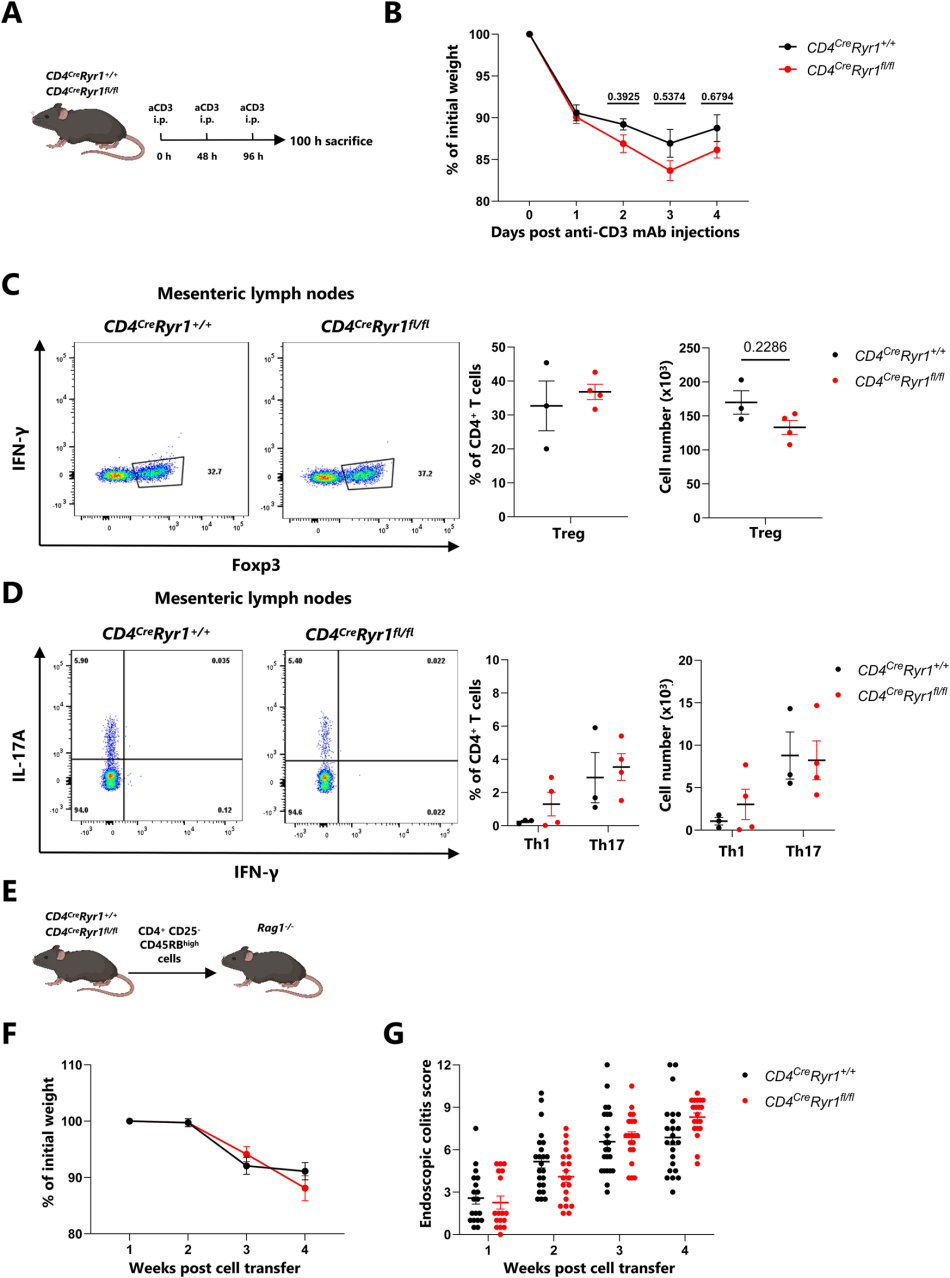
T‐cell‐specific deletion of *Ryr1* does not alter T‐cell‐mediated acute or chronic intestinal inflammation. (A) Schematic of the anti‐CD3 mAb‐induced intestinal inflammation model. *CD4^cre^Ryr1* ^+/+^ and *CD4^cre^Ryr1^fl^* ^/^ *^fl^* mice were injected with anti‐CD3 mAb on Days 0, 2, and 4, and the mice were sacrificed 4 h after the final injection. (B) Weight loss curves in *CD4^cre^Ryr1* ^+/+^ and *CD4^cre^Ryr1^fl^* ^/^ *^fl^* mice during anti‐CD3 mAb‐induced small intestinal inflammation (*n* = 6–7 mice in each group, two independent experiments). (C, D) Representative dot plots and quantification of (C) Foxp3^+^ Treg cell frequency and absolute number and (D) IFN‐γ^+^ Th1 and IL‐17A^+^ Th17 cell frequencies and absolute numbers in mesenteric lymph nodes from anti‐CD3 mAb‐treated *CD4^cre^Ryr1* ^+/+^ and *CD4^cre^Ryr1^fl^* ^/^ *^fl^* mice after 4 h of restimulation with PMA and ionomycin (*n* = 3–4 mice in each group, one experiment). (E) Schematic of the adoptive transfer colitis model. FACS‐sorted CD45RB^high^CD4^+^ T cells from *CD4^cre^Ryr1* ^+/+^ and *CD4^cre^Ryr1^fl^* ^/^ *^fl^* mice were transferred to *Rag1* ^−/−^ recipient mice (*n* = 43 total, 20–23 mice in each group, three independent experiments). (F, G) Weight loss and colonoscopy colitis scores of *Rag1* ^−/−^ mice following CD45RB^high^CD4^+^ T‐cell transfer. All data are presented as the mean ± SEM. Two‐way repeated‐measures ANOVA with Bonferroni post hoc correction was used for weight loss and colonoscopy scores (B, F, G), and the Mann‒Whitney *U* test was used for flow cytometry comparisons (C, D). Each dot represents an individual mouse.

Following anti‐CD3 mAb injection, both *CD4^cre^Ryr1* ^+/+^ and *CD4^cre^Ryr1^fl^* ^/^ *^fl^* mice exhibited rapid weight loss due to a systemic inflammatory response and diarrhea. The extent of weight loss was comparable between the groups, with an average of 15%, revealing similar levels of inflammation in both genotypes (Figure 5A,B). In line with these data, flow cytometric analysis of mesenteric lymph nodes showed no significant differences in the frequencies and absolute cell numbers of Th1, Th17, and Foxp3^+^ Treg cells (Figure 5C,D).

Since anti‐CD3 mAb administration induces acute inflammation, this model is more suitable for studying T‐cell activation and differentiation in vivo, but does not mimic the chronic inflammation observed in IBD. Moreover, *Ryr1* expression is elevated in effector T cells, and the short duration of the anti‐CD3 mAb model may not sufficiently capture the impact of *Ryr1* deletion on their function.

To address this, we employed an adoptive transfer colitis model, which is characterized by chronic intestinal inflammation. In this system, naïve CD45RB^high^CD4^+^ T cells are transferred into *Rag1* ^−^ *^/^* ^−^ mice, which lack functional T and B cells. Upon transfer, the cells predominantly differentiate into Th1 and Th17 effector cells, while conversion into Treg cells is limited. This imbalance makes the model a valuable tool to study the immunopathogenesis of IBD in mice [49, 50]. Regardless of the *Ryr1* genotype of donor T cells, both groups developed colitis with similar colitis scores and weight loss, showing that *Ryr1* expression in CD4^+^ T cells is dispensable for disease progression and severity in this chronic inflammatory model (Figure 5E–G).

### 2.7 *Ryr1* Expression in Th1 and Th17 Cells Is Dispensable for T‐Cell‐Mediated Colitis Progression

While the transfer of CD45RB^high^CD4^+^ T cells mimics chronic T‐cell‐driven colitis through the activation and differentiation of naïve T cells, it does not allow for a direct assessment of RYR1's role in specific effector T‐cell subsets, where its expression is more pronounced. Given that *Ryr1* expression is higher in effector T cells than in naïve T cells, especially in Th1 cells, and that both Th1 and Th17 cells are important mediators of IBDs [51, 52, 53], we next focused on these T‐cell subsets.

To assess whether *Ryr1* deficiency in Th1 and Th17 cells influences colitis pathogenesis, we used a model of effector T‐cell transfer colitis. *CD4^cre^Ryr1* ^+/+^ and *CD4^cre^Ryr1^fl^* ^/^ *^fl^* mice backcrossed to the *IFN‐γ^katushka^IL‐17^eGFP^Foxp3^RFP^* reporter line were used for these experiments.

**FIGURE 6:**
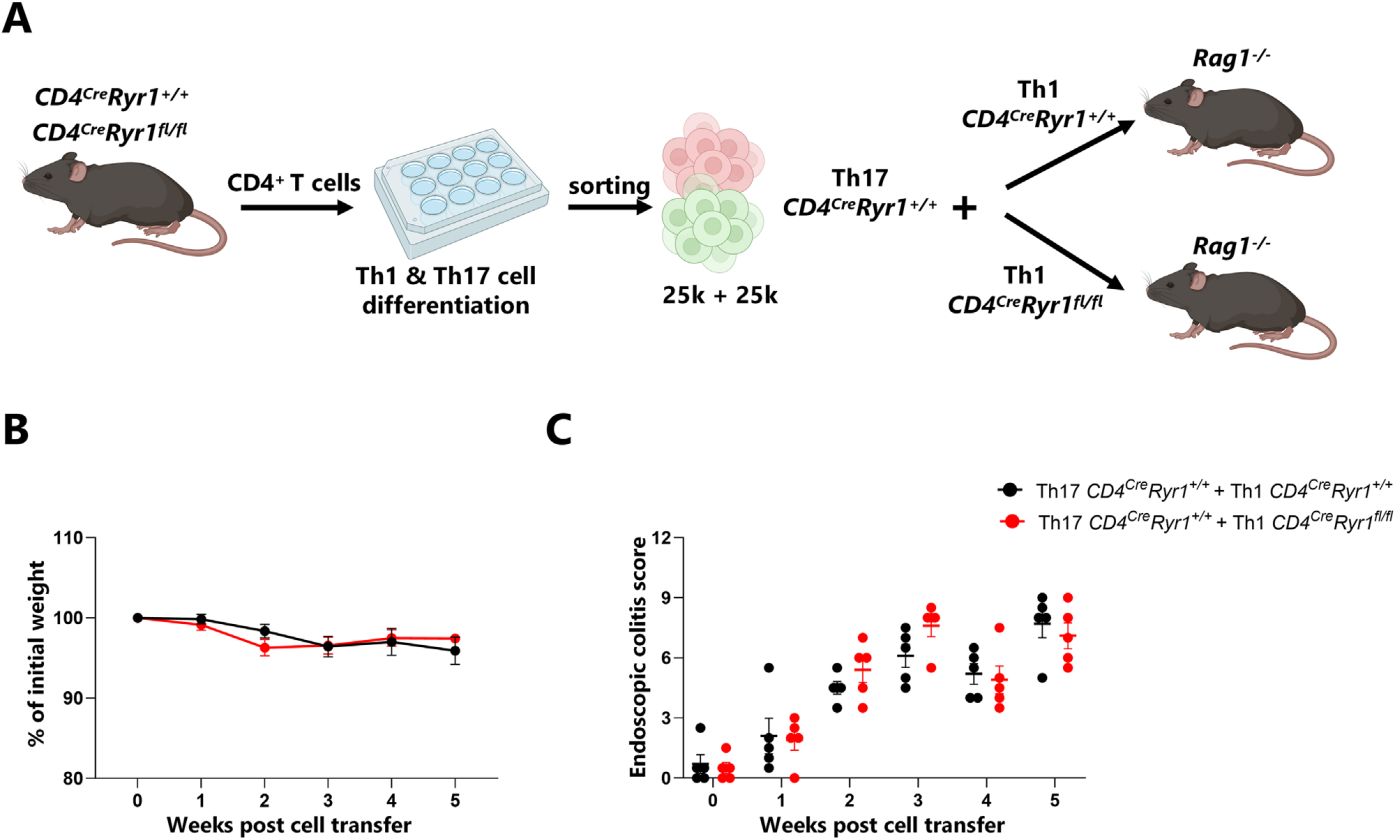
*Ryr1* absence in Th1 cells does not affect colon inflammation severity in effector cell transfer colitis. (A) Schematic representation of Th17 + Th1 cell cotransfer colitis. CD4^+^ T cells were isolated from *CD4^cre^Ryr1* ^+/+^ and *CD4^cre^Ryr1^fl^* ^/^ *^fl^ Foxp3^RFP^IL‐17A^eGFP^IFN‐γ^Katushka^* triple reporter mice. CD4^+^ T cells from *CD4^cre^Ryr1* ^+/+^ mice were differentiated in vitro into Th1 and Th17 cells. CD4^+^ T cells from *CD4^cre^Ryr1^fl^* ^/^ *^fl^* mice were differentiated exclusively into Th1 cells. Th1 (IFN‐γ^+^) and Th17 (IL‐17A^+^) cells were sorted by FACS. A total of 2.5 × 10^4^ *CD4^cre^Ryr1* ^+/+^ Th17 cells combined with 2.5 × 10^4^ Th1 cells from either *CD4^cre^Ryr1* ^+/+^ or *CD4^cre^Ryr1^fl^* ^/^ *^fl^* mice were transferred into *Rag1* ^−/−^ recipient mice (*n* = 10 total, 5 in each group, one experiment). (B) Weekly weight loss and (C) endoscopic colitis scores. All data are presented as the mean ± SEM. Statistical comparisons were made using two‐way repeated‐measures ANOVA with Bonferroni post hoc correction. Each dot represents an individual mouse.

To examine the role of RYR1 in Th1 cells, we cotransferred FACS‐sorted in vitro differentiated wild‐type Foxp3^−^ Th17 cells in combination with either wild‐type or *Ryr1*‐deficient Foxp3^−^ Th1 cells into *Rag1* ^−^ *^/^* ^−^ recipient mice (Figure 6A). This approach ensured robust colitis induction while specifically testing the role of RYR1‐mediated Ca^2+^ signaling in transferred Th1 cells. Colitis developed 1–2 weeks after cell transfer. Colitis severity, assessed by weight loss and weekly endoscopic scoring, revealed that the presence or absence of RYR1 in Th1 cells did not alter the pathogenicity of Th17 cells (Figure 6B,C). These findings indicate that *Ryr1* deficiency in transferred Th1 cells does not change the progression or severity of colitis when cotransferred with wild‐type Th17 cells, suggesting that RYR1 is dispensable for the function of transferred Th1 cells in this model.

**FIGURE 7:**
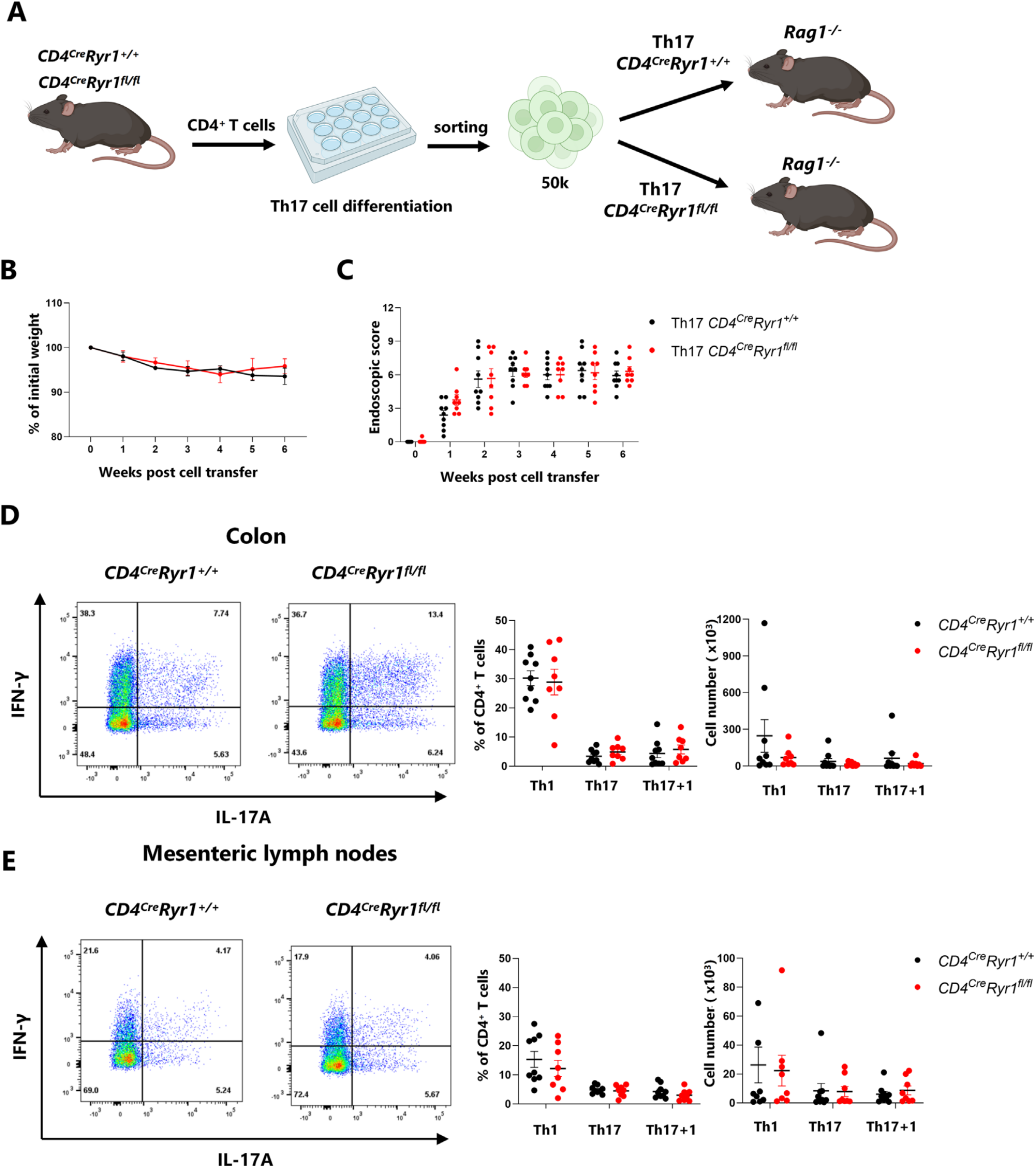
*Ryr1* deficiency in Th17 cells does not affect disease severity in a Th17 adoptive transfer model of colitis. (A) Schematic representation of the Th17 cell transfer colitis model. CD4^+^ T cells were isolated from *CD4^cre^Ryr1* ^+/+^ and *CD4^cre^Ryr1^fl^* ^/^ *^fl^ Foxp3^RFP^IL‐17A^eGFP^IFN‐γ^Katushka^* triple reporter mice and differentiated in vitro into Th17 cells. Th17 (IL‐17A^+^) cells were sorted by FACS, and 5 × 10^4^ *CD4^cre^Ryr1* ^+/+^ or *CD4^cre^Ryr1^fl^* ^/^ *^fl^* Th17 cells were transferred into *Rag1* ^−/−^ mice (*n* = 17 total, 8–9 mice per group, two independent experiments). (B) Weight loss and (C) endoscopic colitis scores at the indicated time points. (D, E) Representative dot plots and quantification of Th1, Th17, and Th1 + Th17 double‐positive cells in the (D) colon and (E) mesenteric lymph nodes are shown as both frequency and absolute number. Mice were sacrificed at Week 6 after transfer. All data are presented as the mean ± SEM. Two‐way repeated‐measures ANOVA with Bonferroni post hoc correction was used for weight loss and colonoscopy scores (B, C); the Mann–Whitney *U* test was used for flow cytometry comparisons (D, E). Each dot represents an individual mouse.

To assess how RYR1 influences the pathogenicity of Th17 cells as well as their transdifferentiation into Th1 cells, we transferred in vitro differentiated Foxp3^−^ Th17 cells from either *CD4^cre^Ryr1* ^+/+^ or *CD4^cre^Ryr1^fl^* ^/^ *^fl^* mice into *Rag1* ^−^ *^/^* ^−^ recipient mice (Figure 7A). Both wild‐type Th17 cells and *Ryr1*‐deficient Th17 cells induced similar levels of intestinal inflammation, as measured by weight loss and endoscopic score (Figure 7B,C). Flow cytometry analysis of T cells isolated from colon and mesenteric lymph nodes showed that a small proportion of T cells still expressed IL‐17A, and a similar proportion expressed both IL‐17A and IFN‐γ, while the majority of T cells expressed IFN‐γ only, suggesting that a major proportion of transferred Th17 cells transdifferentiated into Th1 cells (Figure 7D,E). In the draining lymph nodes, the frequency of Th1 cells was lower in the mice that received *Ryr1*‐deficient cells than in wild‐type controls; however, this difference did not reach statistical significance. No significant differences were observed among T‐cell subset frequencies or absolute cell numbers between wild‐type and *Ryr1*‐deficient Th17 cells. These data indicate that RYR1‐mediated Ca^2+^ signaling is dispensable for Th17 cell‐driven colitis in this model.

**FIGURE 8:**
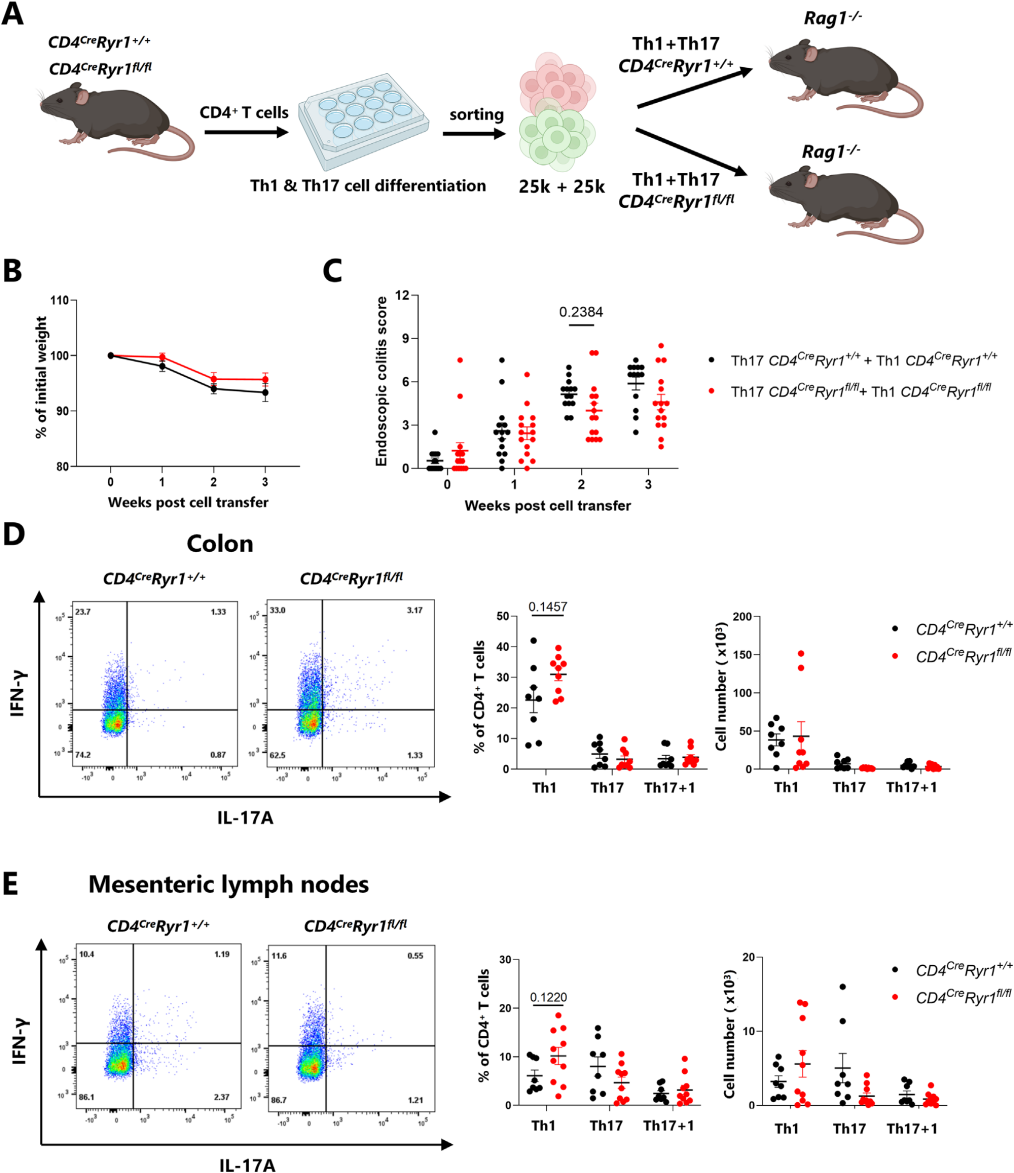
Effect of *Ryr1* deficiency in Th1 and Th17 cells on disease severity in an adoptive transfer colitis model. (A) Schematic representation of the experimental setup. CD4^+^ T cells were isolated from *CD4^cre^Ryr1* ^+/+^ and *CD4^cre^Ryr1^fl^* ^/^ *^fl^ Foxp3^RFP^IL‐17A^eGFP^IFN‐γ^Katushka^* triple reporter mice and differentiated in vitro into Th1 and Th17 cells. A total of 2.5 × 10^4^ FACS‐sorted Th1 (IFN‐γ^+^) and Th17 (IL‐17A^+^) cells were cotransferred into *Rag1* ^−/−^ mice (*n* = 29 total, 14–15 mice per group, four independent experiments). (B) Weight loss and (C) endoscopic colitis scores over the course of the disease (data from four independent experiments). (D and E) Flow cytometric analysis of cells isolated from the colonic mucosa and mesenteric colon‐draining lymph nodes. Representative dot plots and quantification of absolute cell counts and frequencies of Th1, Th17, Th1 + Th17 double‐positive cells, and Tregs (data from three independent experiments) are shown. All data are presented as the mean ± SEM. A two‐way mixed‐effects model (REML) with Šidák's multiple comparisons test was used for weight loss and colonoscopy scores (B, C); the Mann‒Whitney *U* test was used for flow cytometry comparisons (D, E). Each dot represents an individual mouse.

To further investigate the role of RYR1 in colitis mediated by both Th1 and Th17 cells, in vitro polarized wild‐type and *Ryr1*‐deficient Th1 and Th17 cells were sorted by flow cytometry and transferred into *Rag1* ^−^ *^/^* ^−^ recipient mice (Figure 8A). Mice developed signs of colitis, such as colon inflammation, diarrhea, and weight loss, within 2–3 weeks (Figure 8B,C). While there were no significant differences in weight loss or colitis score, we observed a trend toward reduced disease severity in *Rag1* ^−^ *^/^* ^−^ mice reconstituted with *Ryr1*‐deficient Th1 and Th17 cells compared to control mice, both in weight loss and endoscopic colitis score (Figure 8B,C). Flow cytometry analysis of the inflamed colons and the draining lymph nodes showed trends toward a higher frequency of *CD4^cre^Ryr1^fl^* ^/^ *^fl^* Th1 cells, as well as trends toward decreased numbers of *CD4^cre^Ryr1^fl^* ^/^ *^fl^* Th17 cells. However, these findings did not reach statistical significance (Figure 8D,E).

Taken together, these results indicate that *Ryr1* deficiency in Th1 and Th17 cells does not significantly impact colitis development and severity. Thus, RYR1‐mediated Ca^2+^ signaling in Th17 and Th1 cells seems to play a redundant role in intestinal inflammation.

## 3 Discussion

RYR1 has been identified as a key mediator in the formation of Ca^2+^ microdomains within milliseconds of TCR stimulation in CD4^+^ T cells. Previous studies have demonstrated that pharmacological inhibition of ryanodine receptors or disruption of NAADP‐mediated Ca^2+^ signaling impairs CD4^+^ T‐cell activation, proliferation, and differentiation [13, 17, 18, 19, 20]. In this study, we generated and validated a *CD4^cre^Ryr1^fl^* ^/^ *^fl^* conditional knockout mouse line in which *Ryr1* is deleted in αβ T cells. Single‐cell imaging of intracellular Ca^2+^ dynamics following TCR stimulation confirmed the critical role of RYR1 in the formation of Ca^2+^ microdomains within seconds after TCR stimulation. A lower number of initial Ca^2+^ microdomains in *Ryr1‐*deficient cells led to a longer latency to peak Ca^2+^ levels and consequently to reduced overall Ca^2+^ influx. These results confirmed the findings obtained with Jurkat cells with knockdown of *RYR1* and with *Ryr1* ^−^ *^/^* ^−^ murine T cells from fetal liver chimeras [7, 12]. Of note, in the pure population of naïve CD4^+^ T cells used here, deletion of *Ryr1* did not affect proliferation or differentiation in vitro or in vivo *upon* polyclonal stimulation. These results confirm earlier studies in naïve and memory rat T cells in which a > 10‐fold higher concentration of NAADP antagonist BZ194 was required to inhibit proliferation compared to autoantigen‐experienced effector T cells, reflecting the fact that RYR1 was significantly more highly expressed in effector T cells than in naïve T cells [18].

Early models proposed that TCR‐dependent Ca^2+^ signaling is initiated by the formation of localized Ca^2+^ signals generated through NAADP‐mediated release of Ca^2+^ from the endoplasmic reticulum via RYR1 [7]. Recent evidence has revealed the complexity of the NAADP signaling network, identifying two‐pore channels 1 and 2, along with accessory NAADP binding proteins, as its additional components. Specifically, NAADP binding to HN1L/JPT2 facilitated Ca^2+^ release via both RYR1 and TPC1, while its interaction with LSM12 primarily promoted Ca^2+^ release through TPC2. Nevertheless, these broader models suggest that NAADP functions as a versatile second messenger that regulates Ca^2+^ signaling through multiple channels, providing redundancy among specific calcium channels in T cells and enabling possible compensatory mechanisms within this pathway [40, 54, 55]. Moreover, ryanodine receptors, like IP_3_ receptors, are activated by Ca^2+^ through the process of calcium‐induced calcium release [56]. Studies in skeletal muscle cells isolated from myopathy patients with impaired RYR1 function and mouse muscle cells in which RYR1 levels were reduced using siRNA have shown that impaired RYR1 function increased IP_3_Rs expression [57]. Whether similar compensatory mechanisms occur in T cells remains to be investigated. Notably, in some other cell types, Ca^2+^ release from TPC2 has been shown to potentiate IP_3_R‐mediated Ca^2+^ release at ER‐lysosome membrane contact sites. Such spatial coupling between lysosomal TPC2 and ER IP_3_Rs may represent an additional source of redundancy in ER‐release Ca^2+^ signaling [58]. In addition, it is important to consider that antagonists of ryanodine receptors or NAADP‐mediated Ca^2+^ signaling act within hours, leading to impaired T‐cell activation and proliferation and potentially not allowing sufficient time for cells to activate compensatory pathways. In contrast, in the case of the conditional deletion of *Ryr1*, cells might upregulate alternative Ca^2+^ signaling pathways or channels, thereby compensating for the absence of RYR1 and preserving normal activation and proliferative capacity.

RYR1 plays a critical role in striated muscle function, and complete knockout results in perinatal lethality [22]. To investigate the role of *Ryr1* in vivo, Lécuyer et al. generated fetal liver chimeras in which hematopoietic cells lacked RYR1 (unpublished data). In this setting, Ca^2+^ signaling via ryanodine receptors plays a relevant role in T cells in EAE in vivo. Fetal liver chimeras with hematopoietic cells deficient in *Ryr1* had decreased disease activity compared with wild‐type controls. A lower frequency of CD25^+^ and CD69^+^ T cells in the spinal cord in wild‐type fetal liver chimeras was associated with milder disease activity. Moreover, this phenotype was reproduced in conditional knockout *CD4^cre^Ryr1^fl^* ^/^ *^fl^* mice used in our study, demonstrating that Ca^2+^ signaling via RYR1 in T cells is relevant for tissue damage in the context of neuroinflammation.

In contrast, our findings reveal that although RYR1 plays a critical role in TCR‐dependent Ca^2+^ signaling, it is dispensable for CD4^+^ T‐cell function in intestinal inflammation. To investigate this, either anti‐CD3 antibodies were systemically administered to *CD4^cre^Ryr1^fl^* ^/^ *^fl^* mice to induce a cytokine storm and acute intestinal inflammation, or naïve or effector CD4^+^ T cells from *CD4^cre^Ryr1^fl^* ^/^ *^fl^* mice were transferred to *Rag1* ^−/−^ mice, resulting in colitis. These findings may reflect a disease‐specific role of ryanodine receptor–mediated Ca^2+^ signaling. Both intestinal models differ significantly from EAE, primarily in their mode of T‐cell activation. In EAE, the response to a self‐protein of the CNS is restricted by major obstacles. In contrast to the colitis models, few T cells in the periphery are specific for the MOG peptide used for the induction of EAE, and these T cells are under the control of peripheral tolerance mechanisms. In addition, immune cell access to the CNS is limited by the blood‒brain barrier. The extent of CNS inflammation is also determined not only by T‐cell infiltration but also by T‐cell reactivation strength within the CNS [59]. In the anti‐CD3 model, T‐cell stimulation is polyclonal, leading to unspecific activation of T cells, including memory and effector T cells, regardless of their activation threshold and antigen specificity. This can override the physiological antigen presentation and costimulation process, where RYR1‐mediated Ca^2+^ signaling might play a modulatory role. Similarly, in T‐cell transfer colitis, transferred T cells are exposed to a wide variety of microbial antigens in the intestine, which leads to pathogenic T‐cell activation in the absence of Treg cell control. This diverse and high‐antigen‐load environment might be sufficiently potent to trigger T‐cell activation in the absence of RYR1, thereby abrogating the requirement for RYR1‐mediated Ca^2+^ release in T cells in this context. These factors suggest that EAE relies more on cell‐intrinsic modulation of TCR signaling than intestinal inflammation induced by anti‐CD3 or adoptive T‐cell transfer.

In conclusion, our findings suggest that RYR1 has a redundant function in naïve CD4^+^ T‐cell activation, proliferation, and differentiation in vitro, as well as in both acute and chronic models of T‐cell‐mediated intestinal inflammation in vivo. These results contrast with the previously reported roles of RYR1 in neural inflammation, highlighting a potential disease‐specific function of RYR1 in modulating immune responses. Therefore, further studies are warranted to uncover the underlying mechanisms explaining the different roles of RYR1 signaling in CD4^+^ T cells in different diseases.

## 4 Materials and Methods

### 4.1 Mice

*Rag1* ^−/−^ mice were purchased from Jackson Laboratories. The following reporter mice were used: IFN‐γ^Katushka^ [38], IL‐17A^eGFP^ [29], and Foxp3^RFP^ [39]. The mice were kept under specific pathogen‐free (SPF) conditions at the University Medical Center Hamburg‐Eppendorf. All animal procedures were approved by the review board of the City of Hamburg (Behörde für Justiz und Verbraucherschutz der Freien und Hansestadt Hamburg). Age‐ and sex‐matched littermates between 7 and 30 weeks were used.

### 4.2 Generation of *Ryr1* Conditional Knockout Strain

To generate a conditional *Ryr1* allele, we used a conditional ready (*Ryr1* ^1a(EUCOMM)Hmgu^) construct from EUCOMM, in which exon 8 of the mouse *Ryr1* gene is flanked by loxP sites [60]. The L1L2_Bact_P cassette was inserted into the critical exon(s) (Build GRCm39). The cassette was composed of an FRT site followed by a lacZ sequence and a loxP site. This first loxP site is followed by a neomycin resistance gene under the control of the human β‐actin promoter, SV40 polyA, a second FRT site, and a second loxP site. A third loxP site is inserted downstream of exon 8 (Figure 1A). This entire construction functions as a selection cassette. To excise the selection cassette and generate a floxed *Ryr1* allele (*Ryr1* ^1c(EUCOMM)Hmgu^), *Ryr1* ^1a(EUCOMM)Hmgu^ mice were crossed with a Flp deleter line [61]. The resulting conditional *Ryr1* knockout line *Ryr1* ^1c(EUCOMM)Hmgu^ was then crossed with the CD4^cre^ (B6·Cg‐Tg(Cd4‐cre)1Cwi) line [62], referred to in the text as *CD4^cre^Ryr1^fl^* ^/^ *^fl^*.

### 4.3 Isolation of Spleen, Lymph Nodes, and Intestinal Leukocytes

Cells from the spleen and lymph nodes were collected by pressing the organs through 100 µm strainers, followed by filtration through 40 µm strainers. The colon was gently separated from the surrounding mesentery tissue, opened longitudinally, and washed in cold phosphate‐buffered saline (PBS). It was then cut into small pieces and incubated two times in DTT buffer (containing 10% 10X Hank's balanced salt solution, 10% 10X HEPES‐bicarbonate buffer, 5% fetal bovine serum [FBS], and 5 mM ethylenediaminetetraacetic acid [EDTA] in water) at 37°C for 20 min with shaking. Next, the colon pieces were strained through a mesh into gentleMACS Dissociator C tubes (Miltenyi Biotec, Bergisch‐Gladbach, Germany), in which intraepithelial lymphocytes (IELs) were collected by centrifugation, the supernatant was discarded, and the remaining colon pieces were incubated at 37°C for 30 min in collagenase solution (10% FBS, 1% 100X HGPG [HEPES, l‐glutamine, penicillin/streptomycin, and gentamycin], 1 mM CaCl_2_, 1 mM MgCl_2_, 100 U/mL collagenase, 10 U/mL DNAse in RPMI‐1640 medium). The C tubes were then attached upside down onto the sleeve of the gentleMACS Dissociator to obtain lamina propria lymphocytes (LPLs). The digested colon pieces were strained through a mesh strainer and added to the IEL fraction. LPLs and IELs were enriched by using a 67%–40% (two‐phase) Percoll gradient or 40% (one‐phase) Percoll centrifugation by collecting the interphase or cell pellet, respectively.

### 4.4 T‐Cell Isolation

Total CD4^+^ cells were enriched using magnetic‐activated cell sorting (MACS‐Miltenyi Biotec) via positive selection, while naïve CD4^+^ T cells were enriched by negative selection using the EasySep Mouse Naïve CD4^+^ T‐cell isolation kit (STEMCELL Technologies, Vancouver, Canada) according to the manufacturers' instructions. The cell purity exceeded 95% for total CD4^+^ cells and 86% for naïve CD4^+^ T cells, as measured by flow cytometry.

### 4.5 In Vitro CD4^+^ T‐Cell Differentiation

CD4^+^ CD62L^+^CD44^low^ naïve T cells were sorted using FACS from enriched total CD4^+^ T cells (as described above). For each differentiation condition, the cells were cultured in a 96‐well plate at 1 × 10^5^ or 2 × 10^5^ cells per well in 200 µL of full Click's medium (10% FBS, 50 U/mL penicillin, 50 µg/mL streptomycin, 2 mM l‐glutamine, and 0.05 mM β‐mercaptoethanol) supplemented with the following cytokines and antibodies for 4 days. For differentiation of Th1 cells, naïve CD4^+^ T cells were cultured in the presence of 100 U/mL mIL‐2, 10 ng/mL mIL‐12, 10 µg/mL anti‐IL‐4 mAb (clone: 11B11), and 2 µg/mL anti‐CD28 mAb (clone: 37.51) in plates coated with 10 µg/mL anti‐CD3 mAb (clone: 145‐2C11). For the differentiation of Th17 cells, naïve CD4^+^ T cells were cultured in the presence of 10 ng/mL mIL‐6 and 0.25 ng/mL hTGF‐β1, 10 µg/mL anti‐IL‐4 mAb, 10 µg/mL anti‐IFN‐γ (clone: XMG1.2) mAb, and 2 µg/mL anti‐CD28 mAb in plates coated with 10 µg/mL anti‐CD3 mAb. For differentiation of Treg cells, naïve CD4^+^ T cells were cultured in the presence of 50 U/mL mIL‐2 and 2 ng/mL hTGF‐β1 and 2 µg/mL anti‐CD28 mAb in plates coated with 2 µg/mL anti‐CD3 mAb.

We obtained murine IL‐2, IL‐6, and IL‐12 from Miltenyi Biotec and human TGF‐β1 from BioLegend (San Diego, CA, USA). The anti‐mouse CD3 mAb, anti‐mouse CD28 mAb, anti‐mouse IFN‐γ mAb, and anti‐mouse IL‐4 mAb were produced in‐house.

### 4.6 In Vitro CD4^+^ T‐Cell Activation and Proliferation

Naïve CD4^+^ T cells isolated using the EasySep Mouse Naïve CD4^+^ T‐cell isolation kit were stimulated with 2 µg/mL plate‐bound anti‐CD3 and 1 µg/mL soluble anti‐CD28 for 4 and 16 h. T‐cell activation was measured by flow cytometric analysis of NUR77 and IRF4.

For measurement of T‐cell proliferation, EasySep‐purified naïve CD4^+^ T cells were loaded with 2 mM CellTrace violet stain (Invitrogen Thermo Fisher Scientific, Carlsbad, CA, USA) and stimulated with 2 µg/mL plate‐bound anti‐CD3 and 1 µg/mL soluble anti‐CD28 for 48 h. Proliferation was determined by dye dilution via flow cytometry.

For the dose‒response experiments in the supplementary material (Figure S1), naïve CD4^+^ T cells were stimulated with the indicated concentrations of soluble anti‐CD3 in the presence of APCs. NUR77 and IRF4 expression and proliferation were analyzed after 4, 24, and 72 h, respectively, as described above.

### 4.7 Immunofluorescence Staining

Immunofluorescence of RYRs in primary T cells was performed according to [63]. In brief, freshly isolated WT and *Ryr1* ^−^ *^/^* ^−^ naïve CD4^+^ T cells were seeded on slides coated with poly‐l‐lysine (0.1 mg/mL). The cells were fixed with 4% (w/v) *para*‐formaldehyde for 15 min and permeabilized with 0.05% (v/v) saponin for 15 min. To block nonspecific binding sites, cells were incubated with 10% (v/v) FBS overnight at 4°C. Primary mouse anti‐RYR (1:100, 34C, GTX22868, GeneTex) was diluted in 3% (v/v) FBS and incubated overnight at 4°C. The secondary antibody (anti‐mouse Alexa Fluor 488, 1:400, A21202; Thermo Fisher Scientific) was diluted in 3% (v/v) FBS and incubated for 1 h at room temperature. For nuclear staining, the cells were incubated with 4′,6‐diamidino‐2‐phenylindole (DAPI; 62247, Thermo Fisher Scientific, 1:1000) for 10 min at room temperature (RT). Coverslips were mounted with Abberior Mount Solid (Abberior) overnight at 4°C. Images were acquired using a superresolution spinning disc microscope (Visitron) equipped with a CSU‐W1 SoRa (superresolution via optical reassignment) optic (2.8×, Yokogawa), a 100× magnification objective (Zeiss), and a scientific complementary metal‒oxide‒semiconductor camera (Orca‐Flash 4.0, C13440‐20CU, Hamamatsu) in SoRa mode (280×). The following lasers and filters were used for the respective dyes and fluorophores: DAPI, excitation 405‐nm laser and emission 460/50‐nm filter; Alexa Fluor 488, excitation 488‐nm laser and emission 525/50 nm.

### 4.8 Flow Cytometry

For extracellular staining, cells were resuspended in 100 µL of PBS containing live/dead staining dye (Pacific Orange succinimidyl ester, triethylammonium salt, Invitrogen Thermo Fisher) for 10 min at RT. After washing, the cells were stained with fluorochrome‐conjugated antibodies in PBS containing 1% FBS for 10 min at 4°C.

For intracellular staining, cells were restimulated for 4 h with PMA (50 ng/mL, Sigma‐Aldrich, St. Louis, MO, USA), ionomycin (1 mM, Sigma‐Aldrich), and monensin (2 µM, BioLegend) for 4 h at 37°C. After restimulation, extracellular staining was performed as described above. Then, the cells were fixed in 10% formaldehyde for 10 min in the dark and at RT, permeabilized with 0.1% NP‐40 (5 min at RT and dark), and stained intracellularly for 1 h.

The BD LSRFortessa and the FACSAria Fusion/Illu (BD Biosciences, San Jose, CA, USA) were used for cell analysis and cell sorting, respectively.

Extracellular antibodies: anti‐CD45 mAb (clone: I3/2.3, AF700), anti‐CD3 mAb (clone: 17A2, BV421, BV650, BV785), anti‐CD4 mAb (clone: GK1.5, BV785, clone: RM4‐5, PE‐Cy7), anti‐CD8α mAb (clone: 53–6.7, BV650, PE‐Cy7), anti‐CD62L mAb (clone: MEL‐14, APC, BV510), CD44 mAb (clone: IM7, APC‐Cy7).

The following intracellular antibodies were used: anti‐IL‐17A mAb (clone: TC11‐18H10.1, AF‐488), anti‐IL‐10 mAb (clone: JES5‐16E3, PE, PE‐Cy7), anti‐TNF‐α mAb (clone: MP6‐XT22, APC, BV421), anti‐IFN‐γ mAb (clone: XMG1.2, BV711, BV785), anti‐NUR77 mAb (clone:12.14, PE), and anti‐IRF4 mAb (clone: 3E4, PE‐Cy7). All antibodies used were purchased from BioLegend.

### 4.9 Gating Strategy

For every flow cytometry analysis, cells were first gated on FSC‐A versus SSC‐A to select lymphocytes with moderate size (FSC‐A) and low granularity (SSC‐A). Singlets were then gated using FSC‐A versus FSC‐H. For ex vivo analyses, live CD45^+^ leukocytes were identified by excluding dead cells (PacOrange^+^) and gating on CD45^+^ cells, followed by selection of CD3^+^CD4^+^ T cells. For in vitro assays, viable cells were similarly identified by exclusion of PacOrange^+^ events, and CD4^+^ T cells were gated directly.

A representative gating strategy for ex vivo flow cytometric analysis of colonic CD4^+^ T‐cell subsets is shown in Figure S2.

### 4.10 Transfer Colitis and Colonoscopy

For naïve T‐cell transfer colitis, CD45^+^RB^high^CD4^+^ T cells were sorted using FACS, and a total of 200 × 10^3^ cells were injected intraperitoneally (ip) into *Rag1* ^−^ *^/^* ^−^ mice.

For the cytokine‐polarized T‐cell transfer model, total CD4^+^ T cells from *CD4^cre^Ryr1* ^+/+^ *IFN‐γ^katushka^ IL‐17^eGFP^Foxp3^RFP^* (*CD4^cre^Ryr1* ^+/+^) and *CD4^cre^Ryr1^fl^* ^/^ *^fl^ IFN‐γ^katushka^ IL‐17^eGFP^Foxp3^RFP^* (*CD4^cre^Ryr1^fl^* ^/^ *^fl^*) mice were polarized in vitro under Th17 conditions (10 ng/mL mIL‐6, 0.25 ng/mL hTGF‐β1, 10 ng/mL mIL‐1β, 20 ng/mL mIL‐23, 10 µg/mL anti‐IL4 mAb, 10 µg/mL anti‐IFN‐γ mAb, and 2 µg/mL anti‐CD28 mAb in plates coated with 10 µg/mL anti‐CD3 mAb) and Th1 conditions (100 U/mL mIL‐2, 10 ng/mL mIL‐12 and 10 µg/mL anti‐IL4 mAb, and 2 µg/mL anti‐CD28 mAb in plates coated with 10 µg/mL anti‐CD3 mAb). After 4 days, Th17 (IL‐17A^eGFP+^) and Th1 (IFN‐γ^Katushka+^) cells were sorted by FACS. Either Th17 cells alone (5 × 10^4^) or a 1:1 mixture of Th17 cells with Th1 cells (each subset 2.5 × 10^4^) were injected i.p. into *Rag1* ^−^ *^/^* ^−^ mice. Mice were assessed weekly for colitis development and severity by endoscopy (custom‐made colonoscopy system, Karl Storz, Tuttlingen, Germany) as previously described [64, 65]. Mice were anesthetized with isoflurane and scored based on stool consistency, granularity of the mucosal surface, vascularity changes, and colon wall translucency. Each parameter was graded on a scale from 0 to 3, with an overall score ranging from 0 (healthy) to 12 (severe colitis).

### 4.11 Anti‐CD3 mAb‐Induced Intestinal Inflammation

Mice were injected ip with 15 µg of anti‐CD3 mAb (clone: 145‐2C11) at the beginning of the experiment, followed by two injections at 48‐h intervals, and were sacrificed 4 h after the third injection. Mouse weight was measured every day.

### 4.12 Quantitative RT‐PCR

Total CD4^+^ T cells were isolated as described before from the spleens and small intestines of *IFN‐γ^katushka^ IL‐17^eGFP^Foxp3^RFP^* triple reporter mice under steady‐state conditions or anti‐CD3 mAb‐induced inflammatory conditions. For steady‐state analysis, naïve (CD62L^+^CD44^low^), effector (CD62L^−^CD44^high^), and regulatory (Foxp3^+^) CD4^+^ T cells were sorted by FACS. In the inflammation model, mice were treated with anti‐CD3 mAb as described above, and 4 h after the last injection, total CD4^+^ T cells were isolated from the small intestine. Th1 (IFN‐γ^Katushka+^), Th17 (IL‐17A^eGFP+^), and Th1/Th17 cells (IL‐17A^eGFP+^ IFN‐γ^Katushka+^), as well as Foxp3^RFP+^ Treg cells, were sorted using FACS.

RNA was isolated using TRIzol (Life Technologies, Carlsbad, CA, USA) and reverse transcribed using random primers. cDNA concentrations for *Ryr1* and *Hprt* were analyzed by TaqMan real‐time PCR using *Ryr1* and *Hprt* primers (Thermo Fisher Scientific, CA, USA). *Ryr1* values were normalized according to *Hprt* values.

### 4.13 Measurement of Localized Ca^2+^ Signals

A total of 3–5 × 10^6^ EasySep‐isolated naïve CD4^+^ T cells were loaded with Ca^2+^ indicators, 10 µM Fluo‐4 AM, and 20 µM FuraRed AM in RPMI‐1640 with 10% FCS for 20 min in the dark at RT. After 20 min, 2 mL of the same medium was added, and the cells were incubated for an additional 30 min. After washing the cells with Ca^2+^ measurement buffer (140 mM NaCl, 5 mM KCl, 1 mM MgSO_4_, 1 mM CaCl_2_, 20 mM HEPES (pH 7.4), 1 mM NaH_2_PO_4_, 5 mM glucose), the cells were resuspended in the same buffer. For imaging, 10 µL of cells were added to coverslips precoated with bovine serum albumin (5 mg/mL, Sigma‐Aldrich) and poly‐l‐lysine (0.1 mg/mL, Sigma‐Aldrich), which promotes cell adhesion and minimizes movement during measurement. G magnetic beads (10 µm in diameter, Merck Millipore, Burlington, MA, USA) were coated with 0.5 mg/mL anti‐CD3 mAb (clone 145‐2C11, BD Pharmingen BD Biosciences, San Jose, CA, USA) and 0.5 mg/mL anti‐CD28 mAb (clone: 37.51, BD Pharmingen BD Biosciences) for 30–60 min at RT with rotation, then washed and suspended in Ca^2+^ measurement buffer. Imaging was performed using a 100× oil immersion lens on a brightfield light microscope with a xenon arc lamp as a light source and a dual‐view module for both Fluo‐4 and FuraRed signals. After measuring the basal activity for 1 min, anti‐CD3 and anti‐CD28‐coated beads were directly added to the imaging field without disturbing the slide. Ca^2+^ responses were recorded for an additional 2 min. Ca^2+^ microdomains were measured and analyzed as described in [66].

### 4.14 Measurement of Global Ca^2+^ Signals

An aliquot of 10^7^ freshly EasySep‐isolated murine naïve CD4^+^ T cells was loaded with the membrane‐permeable AM ester of Fura‐2 (4 µM) for 15 min at 37°C in 1 mL of full Click's medium. After 15 min, 4 mL of fresh medium was added. The cells were rinsed twice and resuspended in Ca^2+^ measurement buffer. Cells were added to prepared coverslips coated with bovine serum albumin (5 mg/mL, Sigma‐Aldrich) and poly‐l‐lysine (0.1 mg/mL, Sigma‐Aldrich).

Cells were stimulated with 2 µg/mL anti‐CD3 mAb (clone 145‐2C11, BD Pharmingen BD Biosciences) after 20 s of recording. Imaging was performed on a Leica IRBEmicroscope (Wetzlar, Germany) with 40× magnification. A Sutter DG‐4 (Novato, CA, USA) was used as a light source, and frames were acquired with an electron‐multiplying charge‐coupled device camera (Hamamatsu, Hamamatsu, Shizuoka, Japan). One frame every 2 s was acquired using a Fura‐2 filter set (excitation, HC 340/26, HC387/11; beam splitter, 400DCLP; emission, 510/84; all in nanometers; AHF Analysentechnik (Tübingen‐Pfrondorf, Germany)). The intracellular Ca^2+^ concentration was determined in Fura‐2‐loaded murine CD4^+^ T cells. Therefore, *R*_min_ [using the lowest ratio (*R*) and fluorescence (*F*) after EGTA chelation] and R_max_ [using the highest *R* and *F* after ionomycin incubation] of Fura‐2 in single‐cell measurements were assessed.

### 4.15 Statistics

Statistical analyses were performed using GraphPad Prism (GraphPad Software, La Jolla, CA, USA), and flow cytometry data were analyzed with FlowJo software (BD Biosciences). Statistical tests used are specified in the figure legends. *p* values of *p* < 0.05 was considered statistically significant.

## Author Contributions

S.H., H.‐W.M., M.N., and T.B. conceived and supervised the study. S.D.T. and M.N. performed the experiments, analyzed the data, and wrote the manuscript. B.‐P.D. and F.M. provided expertise in Ca^2+^ imaging and data analysis. L.H. carried out the immunofluorescence staining. F.S., Ma.B., and Me.B. assisted with experimental processing. A.G., N.G., D.L., and A.F. provided critical input and feedback on the manuscript. All authors reviewed and approved the final version.

## Funding

This study was supported by the Deutsche Forschungsgemeinschaft: SFB1328 (project number FKZ 335447717), project A03 to S.H. and H.W.M., A01 to A.H.G. and A.F., project A02 to B.P.D., project A14 to N.G.

## Ethics Statement

All animal experiments were approved by the review board of the City of Hamburg (Behörde für Justiz und Verbraucherschutz der Freien und Hansestadt Hamburg; Registration Nos. N 67/20 and N 021/25). No human studies were performed.

## Conflicts of Interest

The authors declare no conflicts of interest.

## Supporting information




**Supporting File**: eji70252‐sup‐0001‐SuppMat.pdf.

## Data Availability

The data supporting the findings of this study are available from the corresponding author upon reasonable request.
