# Supplementary material for: Ryanodine Receptor 1 Is Dispensable for CD4+ T‐Cell Differentiation and Effector Function in Intestinal Inflammation Models
Source: Eur J Immunol. 2026 Aug 17;56(8):e70252. doi: 10.1002/eji.70252 (PMC13482298; doi:10.1002/eji.70252)
Supplement: Supplementary file 1 [file EJI-56-e70252-s001.pdf]

Fig. S1

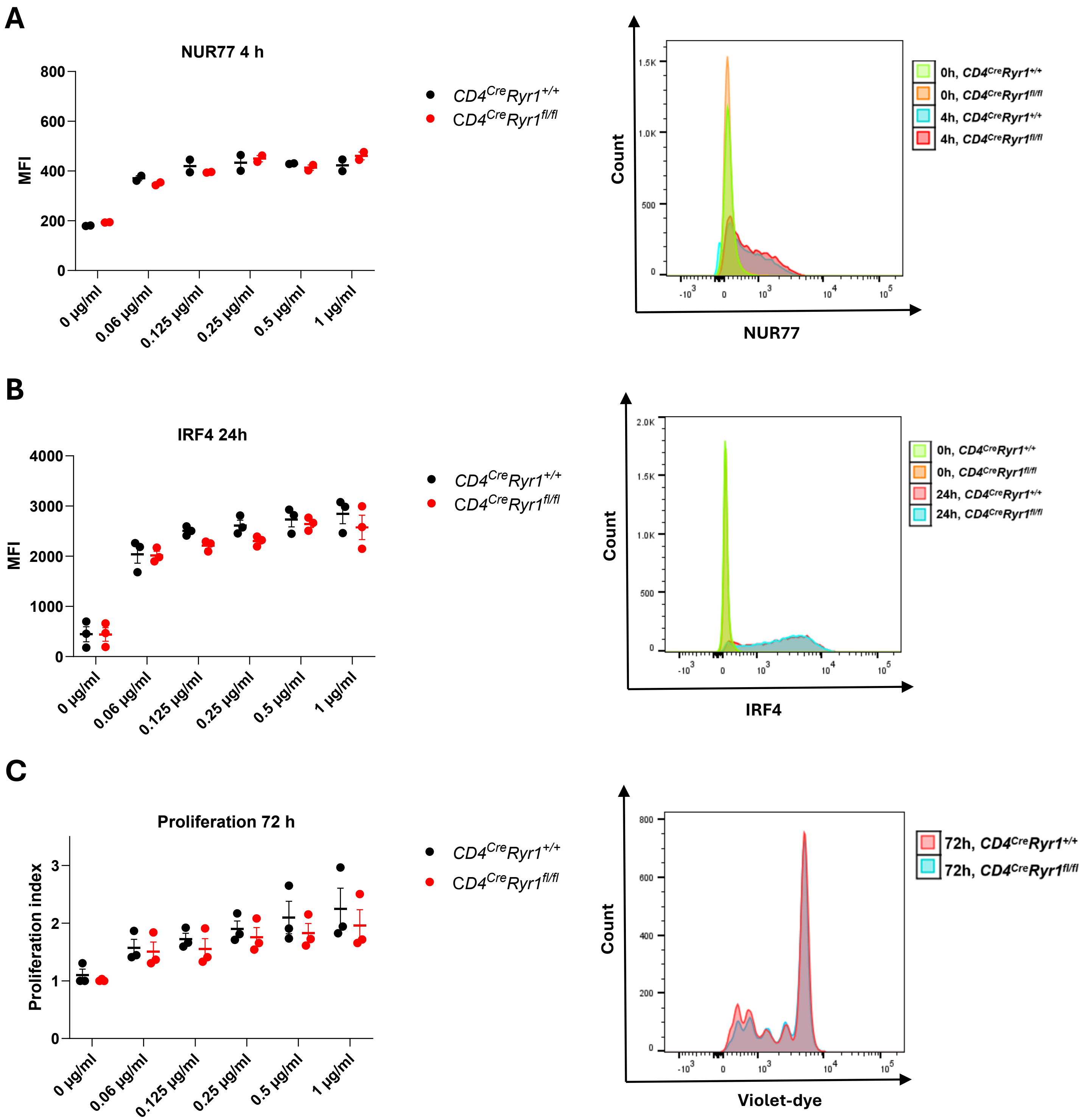

Fig.S1. Anti-CD3 dose-response analysis of naïve CD4$^+$ T cells. Naïve CD4$^+$ T cells isolated from *CD4^{cre}Ryr1$^{+/+}$* or from *CD4^{cre}Ryr1$^{fl/fl}$* mice were stimulated with the indicated concentrations of anti-CD3 antibody in the presence of APCs. **A, B)** NUR77 and IRF4 expression were assessed after 4 h and 24 h of stimulation, respectively. Representative histograms at the highest stimulation concentration (right) and mean fluorescence intensity (MFI, left) are shown. **C)** Naïve CD4$^+$ T cells were labeled with CellTrace Violet before stimulation with anti-CD3 antibody in the presence of APCs. Proliferation index (left) and representative histogram (right) are shown. Each dot represents one biological replicate, and bars indicate mean ± SEM. Groups were compared at each anti-CD3 concentration using unpaired two-tailed Mann–Whitney tests.

Fig. S2

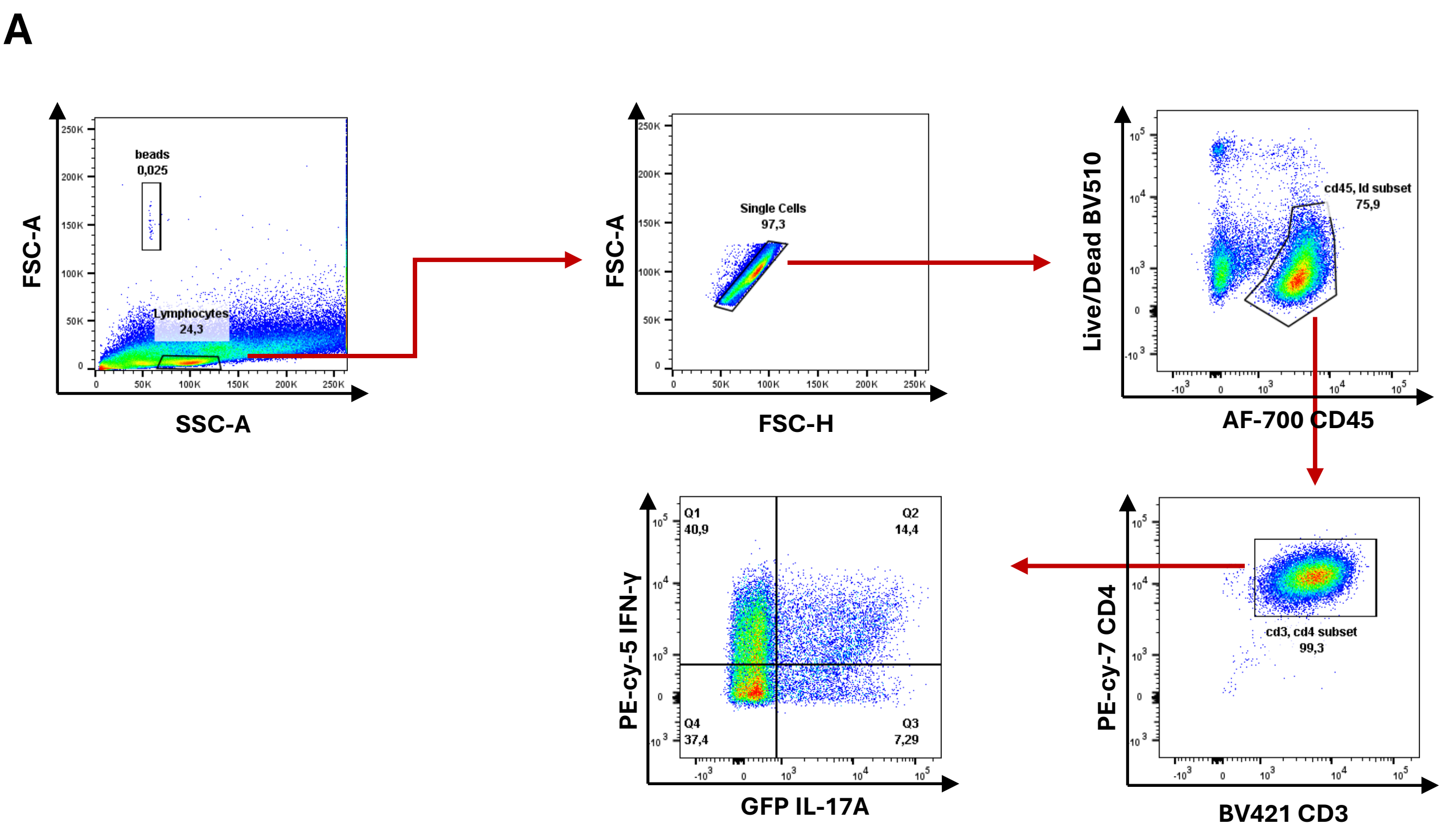

Fig. S2. Gating strategy for identification of CD4⁺ T cell subsets from the colon.
A) Representative flow cytometry plots showing the sequential gating strategy used for *ex vivo* analysis in the Th17 adoptive transfer colitis model (corresponding to Fig. 7). Debris was excluded based on FSC-A and SSC-A characteristics, and single cells were identified by gating on FSC-A and FSC-H. Live $CD45^+$ leukocytes were gated using a viability dye (Live/Dead BV510 vs. AF700 CD45). From this population, $CD3^+CD4^+$ T cells were identified. Within the $CD4^+$ T cell population, IL-$17A^+$ (GFP^+) and IFN-γ^+ (PE-$Cy5^+$) subsets were quantified.
All frequencies and absolute numbers were calculated relative to the total $CD4^+$ T cell population. Representative staining is shown.
